# Differences in psychological treatment outcomes by ethnicity and gender: an analysis of individual patient data

**DOI:** 10.1007/s00127-024-02610-8

**Published:** 2024-02-06

**Authors:** Laura-Louise C. Arundell, Rob Saunders, Joshua E. J. Buckman, Glyn Lewis, Joshua Stott, Satwant Singh, Renuka Jena, Syed Ali Naqvi, Judy Leibowitz, Stephen Pilling

**Affiliations:** 1 CORE Data Lab, Centre for Outcomes Research and Effectiveness, Research Department of Clinical, Educational and Health Psychology, University College London, Gower Street, London, UK; 2 National Collaborating Centre for Mental Health, Royal College of Psychiatrists, London, UK; 3 Division of Psychiatry, University College London, London, W1T 7NF UK; 4 iCope, Camden and Islington Psychological Therapies Services, Camden and Islington NHS Foundation Trust, London, UK; 5 ADAPT Lab, Research Department of Clinical, Educational and Health Psychology, University College London, Gower Street, London, UK; 6 Waltham Forest Talking Therapies, North-East London NHS Foundation Trust, London, UK; 7 North-East London NHS Foundation Trust, London, UK

**Keywords:** Ethnicity, Gender, Anxiety, Depression, Psychological therapy, Outcome measures

## Abstract

**Purpose:**

There are discrepancies in mental health treatment outcomes between ethnic groups, which may differ between genders. NHS Talking Therapies for anxiety and depression provide evidence-based psychological therapies for common mental disorders. This study examines the intersection between ethnicity and gender as factors associated with psychological treatment outcomes. Aims were to explore by gender: (1) differences in psychological treatment outcomes for minoritized ethnic people compared to White-British people, (2) whether differences are observed when controlling for clinical and socio-demographic factors associated with outcomes, and (3) whether organization-level factors moderate differences in outcomes between ethnic groups.

**Methods:**

Patient data from eight NHS Talking Therapies for anxiety and depression services (*n* = 98,063) was used to explore associations between ethnicity and outcomes, using logistic regression. Stratified subsamples were used to separately explore factors associated with outcomes for males and females.

**Results:**

In adjusted analyses, Asian (OR = 0.82 [95% CI 0.78; 0.87], *p* < .001, ‘Other’ (OR = 0.79 [95%CI 0.72–0.87], *p* < .001) and White-other (0.93 [95%CI 0.89–0.97], *p* < .001) ethnic groups were less likely to reliably recover than White-British people. Asian (OR = 1.48 [95% CI 1.35–1.62], *p* < .001), Mixed (OR = 1.18 [95% CI 1.05–1.34], *p* = .008), ‘Other’ (OR = 1.60 [95% CI 1.38–1.84], *p* < .001) and White-other (OR = 1.18 [95% CI 1.09–1.28], *p* < .001) groups were more likely to experience a reliable deterioration in symptoms. Poorer outcomes for these groups were consistent across genders. There was some evidence of interactions between ethnic groups and organization-level factors impacting outcomes, but findings were limited.

**Conclusions:**

Across genders, Asian, ‘Other’ and White-other groups experienced worse treatment outcomes across several measures in adjusted models. Reducing waiting times or offering more treatment sessions might lead to increased engagement and reduced drop-out for some patient groups.

**Supplementary Information:**

The online version contains supplementary material available at 10.1007/s00127-024-02610-8.

## Introduction

People from minoritized ethnic communities in the UK are less likely to receive treatment for common mental disorders (CMDs) despite the prevalence of anxiety and depression being highest for some minoritized ethnic groups [1, 2]. Being racialised as ‘*non-White*’ (i.e. being ascribed a race or ethnic category based on perceptions of skin colour) has been shown to impact how people experience healthcare and how they are treated by a range of services [3, 4]. Whilst experiences differ, people belonging to minoritized groups socially assigned as ‘minorities’ are more likely to report healthcare discrimination [5] and are less likely to receive treatment, experience poorer outcomes [1], and are more likely to be detained and treated under the Mental Health Act [6].

NHS Talking Therapies for anxiety and depression in England (formerly ‘Improving Access to Psychological Therapies’, referred to as ‘IAPT’ hereafter) provide evidence-based psychological therapies for CMDs [7]. IAPT was established to provide equitable access to talking therapies, but 12 years after its initiation, and despite overall increased access [8], some inequity remains. Attempts to resolve ethnic disparities in mental health service use have generally focused on access and engagement discrepancies, and cultural barriers to treatment [9, 10]. Several studies have also highlighted the importance of organization-level factors, such as those that encompass service structure, design, and delivery, which are all associated with outcomes from psychological treatment [11]. Implementing ‘organization-specific’ cultural adaptations to mental health care aimed at people from minoritized ethnic groups may also be associated with better treatment outcomes in a variety of settings [12]. Although implementing cultural adaptations can be challenging, it is likely to be feasible that some form of cultural adaptation is possible in almost every service setting, and whether or not ‘organization-specific’ adaptations are able to be made, there is also a role for ensuring that clinicians are trained to adapt their practices and provide culturally sensitive care [12–14].

Socio-demographic factors, including age, gender, religion and marital or employment status are associated with the incidence of mental health problems, treatment access and outcomes [15–18], and with clinicians’ treatment decisions [19]. Intersections between these factors are associated with further inequalities in mental health; for example men who identify as Muslim and Asian have been found to be less likely to receive treatment in some IAPT services relative to White-British men with other religious identities [20, 21]. Despite some examples of intersectional effects on mental health treatment access and outcomes, this has been largely under-studied [22] and existing studies have tended not to address how ethnicity and gender may intersect to influence outcomes differently between genders [23, 24].

Regarding gender differences, women are more likely to be diagnosed with CMDs than men [25, 26] and women from South Asian, Black African and African-Caribbean communities have the highest incidence of CMDs but are less likely to receive care for them relative to White-British women [8]. Regarding disparities in treatment effectiveness and drop-out between genders, the evidence base is inconclusive; some studies have reported no significant differences between genders, yet others have found the opposite [17, 27].

The current study examines factors associated with psychological treatment outcomes beyond access and engagement, for minoritized ethnic groups of males and females. The study aims to explore (1) observed differences in psychological treatment outcomes for people from minoritized ethnic communities compared to White-British people, (2) whether differences are observed when controlling for clinical and socio-demographic factors known to be associated with outcomes and (3) whether organization-level factors moderate differences in outcomes between ethnic groups.

## Methods

### Participants

Eight IAPT services from the North, Central and East London IAPT Service Improvement and Research Network (NCEL IAPT SIRN) [17] comprised the data. The dataset included 483,683 patients referred to the services between August 2008 and August 2020. Two stratified subsamples of this dataset were used to explore the factors associated with outcomes for patients identifying either as male or female, as well as primary analyses for the full sample regardless of gender. Patients were included in the analysis if they were aged 18 or over when referred, had completed a treatment course (i.e., had an end of treatment date recorded and were not still receiving treatment), were above the clinical cut-off for ‘caseness’ on any of the depression or anxiety measures used at baseline, and provided self-ascribed ethnicity information. After exclusions, 98,063 patients were included in the whole sample, 66,293 in the female subsample and 31,515 in the male sub-sample (see Figure S1 in Supplementary Material).

Neither consent nor ethical approval was required for this study (confirmed by the Health Research Authority July 2020, reference number 81/81C). Data were provided by services as part of the NCEL agreement in accordance with the procedures of the host institution and the NHS Trusts which operate the IAPT services (project reference: 00519-IAPT).

### Categorisations of ethnicity and gender

The term ‘minoritized ethnicity’ is used to refer to people from ethnic groups 1–16 (Table 1) including people often racialised as ‘White’ who do not identify as ‘White-British’, such as ‘Irish’ and ‘White-other’ groups. Including a distinct group for White-other allowed for a comparison of differences in outcomes for people from minoritized groups who might have different experiences on the basis of being perceived as ethnically and culturally different to those identified as ‘White-British’. All ethnicity categories are listed next to the high-level Office for National Statistics (ONS) categories they are included under. Individuals in minoritized ethnic groups were compared against White-British people.

**Table 1:** Descriptions of ethnic group categorisation, routinely collected measures and outcomes

Ethnic group	ONS ethnic group
1. Asian—Bangladeshi	Asian
2. Asian—Indian	
3. Asian—Pakistani	
4. Other Asian	
5. Black—African	Black
6. Black—Caribbean	
7. Other Black	
8. Chinese	Chinese
9. Mixed—White & Black African	Mixed
10. Mixed—White & Black Caribbean	
11. Mixed—White and Asian	
12. Other mixed	
13. Other ethnic group	Other
14. White—other	White*
15. White—any other White background	
16. White—Irish	
17. White—British	

Measures	Description
Patient Health Questionnaire 9-item (PHQ-9) [28]	Used to measure symptoms of depression, scores of 10 or above indicate clinical ‘caseness’ for depression; reduction in scores of 6 or more indicated reliable improvement in symptoms
Generalised Anxiety Disorder Scale 7-item (GAD-7) [29]	Used to measure generalised anxiety symptoms, a score of 8 or higher is used for caseless and a cut-off of 4 is used for reliable improvement
Work and Social Adjustment Scale (WSAS) [30]	Used to measure personal functioning with regard to ‘ability to work’, ‘social activities’, ‘home management’, ‘social activities’, ‘private leisure activities’, and ‘close relationships’; scores range from 0–8
IAPT Phobias Scale [31]	Three questions which assess the degree of avoidance of situations related to phobic anxiety including: agoraphobia, social phobia and specific phobias
Problem descriptor	Based on the ICD-10 code this represents each patient’s probable or confirmed diagnosis in order to match the presenting problem to the right evidence-based treatment (recorded after assessment sessions)
Demographics	Gender (non-binary gender is not collected in the dataset used), age, index of multiple deprivation (IMD—used to measure the relative deprivation of small geographical areas in England. IMD ranks each area from most to least deprived using a weighted composite index score which incorporates income, unemployment, health and disability, crime, education, barriers to housing and the quality of the local environment [32], ethnicity (using the ONS categories outlined in above) (collected at assessment only)
Long-term health conditions (LTCs)	Self-reported presence of any existing long-term physical health condition (collected at assessment only)
Psychotropic medication	Whether or not patients are prescribed psychotropic medication, recorded at each clinical contact
Employment	Whether or not patients were in any employment when they started treatment

Outcomes	Description
Reliable improvement	A patient is considered to have experienced a reliable improvement if a reduction in symptom scores is reported that is above the threshold for the error of measurement on either the PHQ-9 or GAD-7, or an anxiety disorder specific measure [33] (ADSM- used when specific anxiety disorders are recorded as the ‘problem descriptor’ [11, 31], following treatment, and no reliable deterioration is observed—see below)
Reliable recovery	A patient is determined to have experienced reliable recovery if: - they meet criteria for recovery (i.e., moving from above the threshold for caseness for either depression or anxiety on the PHQ-9, GAD-7, or an anxiety disorder specific measure before treatment, to below the threshold for caseness on measures of both depression and anxiety) at the last appointment [33], and - they meet the criteria for reliable improvement [33]
Reliable deterioration	A patient is considered to have experienced a reliable deterioration if they report an increase in symptom scores above the threshold for the error of measurement on any of the symptom-based outcome measures, following treatment, and they have not experienced a reliable improvement [33]
Drop-out	A patient is considered to have dropped out of treatment if the treating clinician records a reason for discharge from the IAPT services that suggests a premature end to the planned treatment episode. The converse being that the treatment was completed. This outcome excludes those referred on for treatment in other services that had fewer than three treatment sessions

*The ONS amalgamates all ‘White’ ethnic sub-groups together, including White-British. For this study, analyses were performed such that ‘White-British’ was separated from other White groups allowing for comparisons between ‘White British’ and other ethnicities including ‘White-non-British’groups

Two stratified sub-samples were used to explore factors associated with outcomes for people self-ascribed as males and females. These are the only options patients had to self-identify their gender at the time data used in this study was collected (IAPT V1.5). It is acknowledged that binary terms of male and female, which more accurately refer to biological sex [34], are often used to refer to gender in research but do not fully encapsulate the ways in which people might choose to describe their gender.

### Measures and outcomes

IAPT services collect outcome measures routinely, at each contact. Table 1 shows the self-report measures and data items collected at assessment, in addition to the outcomes used in this study (adapted from Buckman and colleagues [35]).

### Plan of analysis

The study followed a pre-registered analysis protocol with no deviations [36]. STATA code is available upon request to the corresponding author. The study comprised of the following analyses:

1. Comparison of baseline data between White-British people and people from minoritized ethnic groups using independent samples t-tests for continuous variables, and chi-square tests for categorical variables. The first set of analyses compared outcomes using two binary categories of ethnicity (i.e., a White-British and an aggregate minority ethnic group). This initial set of analyses intended to provide a broad overview of the baseline discrepancies that are observed between groups racialised as ‘*non-White’* compared to the White-British group.
2. Separate logistic regressions to model the association between two binary categories of ethnicity (i.e., a White-British and an aggregate minority ethnic group) and each outcome in turn, whilst controlling for several potential confounding variables. Each set of analyses were run on the whole data sample, and then separately on the male and female sub-samples. Five models were built for each outcome.

Model 1: ethnicity

Model 2: (Model 1) + service

Model 3: (Model 2) + age, gender (for whole sample analyses only), long-term condition (LTC) status

Model 4: (Model 3) + baseline severity PHQ-9, baseline severity GAD-7, baseline severity WSAS, diagnosis/presenting problem, each item from the IAPT-Phobias Scale (social phobia, agoraphobia, specific phobia)

Model 5: (Model 4) + IMD, employment status, psychotropic medication status.

(Detail in supplementary material—appendix 2).

(3) Separate logistic regression models to explore the associations between ethnic group and each outcome, whilst controlling for potential confounders. Each set of analyses was run on the whole data sample, and then on each sub-sample of males and females. Analyses compared outcomes from sub-groups of people from minoritized ethnic groups (i.e., people reported as anything other than White-British according to ONS categories of ethnic group (see Table 1), to outcomes from White-British people using ‘White-British’ as the reference category. Five models (as above) were built for each outcome (see supplementary material—appendix 2).
(4) Regressions with interaction terms were used to explore which organization-level factors [11, 12] might be associated with outcomes for people in different ethnic groups (Table 1). A likelihood ratio test was used to identify whether adjusted models were improved by the inclusion of an interaction term. Each set of analyses were run on the whole analytic data sample, and then on each sub-sample of males and females. Analyses were performed with each outcome measure. Details of the analyses and organization-level variables used are provided in supplementary material—appendix 3).

Analyses above were also performed on stratified samples of males and females (see supplementary material—appendix 3).

## Results

### Comparison of White-British and minoritized ethnicity patients

The analytic data sample comprised 98,063 individuals (68% female). People in the amalgamated minoritized ethnicity group had significantly higher baseline PHQ-9, GAD-7 scores, tended to be younger on average and had longer waiting times compared to the White-British group. A significantly higher proportion of people in the minoritized ethnic group were not employed, presented with long-term physical health conditions and lived in more deprived areas (Table 2).

**Table 2:** Comparison of baseline demographic and clinical information between ethnic groups (whole analytic data sample *n* = 98,063). Differences between White-British and minoritized ethnic groups across outcome measures are also displayed

Ethnic group (ONS)	*N*	%
White-British	45,107	46.00
Asian	11,356	11.58
Black	10,434	10.64
Chinese	706	0.72
Mixed	5773	5.85
Other	3687	3.76
White—other	21,040	21.46
Total	98,063	

Continuous variables	White-British			All minoritized ethnicities			Difference
	*N*	Mean	Sd	*N*	Mean	SD	*p* value
PHQ-9	45,105	14.74	5.58	52,953,	15.95	5.56	< .001
GAD-7	45,104	13.72	4.40	52,934	14.35	4.40	< .001
WSAS 1	37,205	5.35	2.85	45,316	5.71	2.94	< .001
WSAS 2	37,217	3.45	2.38	45,316	3.86	2.48	< .001
WSAS 3	37,216	4.29	2.39	45,309	4.62	2.50	< .001
WSAS 4	37,213	3.52	2.51	45,304	4.02	2.61	< .001
WSAS 5	37,214	3.93	2.43	45,303	2.25	2.50	< .001
Social phobia	44,444	3.02	2.50	51,628	3.46	2.67	< .001
Specific phobia	44,431	2.10	2.65	51,600	2.60	2.85	< .001
Agoraphobia	44,430	2.60	2.68	51,604	2.96	2.81	< .001
Number of LI sessions	45,107	2.89	2.79	52,956	2.93	2.79	.036
Number of HI sessions	45,107	4.79	5.61	52,956	4.40	5.33	< .001
Weeks waited from referral to assessment	45,091	3.66	4.47	52,931	3.91	4.73	< .001
Weeks waited from assessment to treatment	42,120	8.48	8.21	49,216	8.98	8.59	< .001
Age	45,107	38.65	14.60	52,956	37.06	12.51	< .001

Categorical variables	White-British		All minoritized ethnicities		*p* value
	*N*	%	*N*	%	
Employed	34,961	78.34	37,267	70.34	< .001
Unemployed	9669	21.66	14,983	28.68	
Existing LTC	11,598	27.17	13,835	28.00	.006
No existing LTC	31,083	72.83	35,589	72.00	
IMD decile—high	4315	15.21	3341	9.69	< .001
IMD decile—low	24,058	84.80	31,149	90.31	

Outcome measure	White-British	All minorized ethnicities	*X*^*2*^		
Reliably recovered	49%	42%	*X*^*2*^ (1, *N* = 98,063) = 388.09, *p* < .001)		
Deteriorated	7%	8.4%	*X*^*2*^(1,* N* = 98,063) = 90.01, *p* < .001)		
Dropped out	29%	32%	*X*^2^(1,* N* = 98,063) = 90.01, *p* < .001)		

*IMD* indices of multiple deprivation, *HI *high intensity treatment, *LI *low intensity treatment, *WSAS *work and social adjustment scale (items 1–5), *PHQ-9 *patient health questionnaire (depression symptom measure), *GAD-7* generalised anxiety disorder scale (anxiety symptom measure), *IMD *indices of multiple deprivation, *LTC *long-term condition, *ONS *office for national statistics

Differences were further explored using linear regression, where effects of ethnicity were found across treatment outcomes in the unadjusted models, which remained when adjusting for other factors (Table 3).

**Table 3:** Results of logistic linear regression analyses exploring differences in outcomes between the White-British group and an amalgamated minoritized ethnicity group

Model	Variables	Reliable recovery: odds ratio (95% CI) *p-*value	Reliable improvement: odds ratio (95% CI)* p-*value	Reliable deterioration: odds ratio (95% CI) *p-*value	Drop-out: odds ratio (95% CI) *p-*value
Model 1	Ethnicity	1.29 (1.26–1.32) *p* < .001	1.17 (1.14–1.20) *p* < .001	0.79 (0.76–0.83) *p* < .001	0.89 (0.87–0.92) *p* < .001
Model 2	(Model 1) + Service	1.30 (1.27–1.34) *p* < .001	1.17 (1.14–1.20) *p* < .001	0.78 (0.75–0.82) *p* < .001	0.86 (0.83–0.89) *p* < .001
Model 3	(Model 2) + Age, gender, LTC status	1.29 (1.26–1.32) *p* < .001	1.19 (1.15–1.22) *p* < .001	0.80 (0.76–0.84) *p* < .001	0.89 (0.86–0.91) *p* < .001
Model 4	(Model 3) + Baseline severity PHQ9, Baseline severity GAD7, Baseline severity WSAS, diagnosis/presenting problem, social phobia, agoraphobia, specific phobia	1.13 (1.10–1.17) *p* < .001	1.14 (1.10–1.17) *p* < .001	0.77 (0.73–0.82) *p* < .001	0.95 (0.91–0.98) *p* = .002
Model 5	(Model 4) + IMD, employment status, Medication status	1.09 (1.06–1.13) *p* < .001	1.10 (1.07–1.14) *p* < .001	0.80 (0.75–0.85) *p* < .001	0.96 (0.93–0.99) *p* = .021

### Comparison of White-British and minoritized ethnicity patients using ONS categories

Further differences in outcomes by ethnicity were explored using the six ONS ethnicity categories and separating the White category into White-British (used as the reference category) and White-Other (Table 4).

**Table 4:** Results of logistic regression analyses using ONS categories of ethnicity (reference category = White-British)

Model	Variables	ONS ethnicity categories	Reliable recovery: odds ratio (95% CI) *p-*value	Reliable improvement: odds ratio (95% CI) *p-*value	Reliable deterioration: odds ratio (95% CI) *p-*value	Drop-out: odds ratio (95% CI) *p-*value
Model 1	Ethnicity	Asian Black Chinese Mixed Other White-other	0.70 (0.67–0.73) *p* < .001 0.80 (0.76–0.83) *p* < .001 1.06 (0.91–1.23) *p* = .436 0.80 (0.75–0.84) *p* < .001 0.59 (0.55–0.64) *p* < .001 0.83 (0.80–0.85) *p* < .001	0.81 (0.78–0.85) *p* < .001 0.90 (0.86–0.94) *p* < .001 1.00 (0.85–1.18) *p* = .957 0.90 (0.85–0.95) *p* < .001 0.69 (0.64–0.74) *p* < .001 0.88 (0.85–0.91) *p* < .001	1.36 (1.27–1.47) *p* < .001 1.27 (1.18–1.38) *p* < .001 1.15 (0.87–1.51) *p* = .335 1.21 (1.10–1.34) *p* < .001 1.51 (1.35–1.70) *p* < .001 1.17 (1.10–1.24) *p* < .001	1.29 (1.23–1.35) *p* < .001 1.34 (1.28–1.41) *p* < .001 0.80 (0.67–0.96) *p* = .018 1.25 (1.18–1.33) *p* < .001 1.23 (1.14–1.32) *p* < .001 0.91 (0.87–0.94) *p* < .001
Model 2	(Model 1) + Service	Asian Black Chinese Mixed Other White-other	0.68 (0.65–0.71) *p* < .001 0.79 (0.75–0.82) *p* < .001 1.08 (0.93–1.25) *p* = .322 0.79 (0.75–0.84) *p* < .001 0.59 (0.55–0.64) *p* < .001 0.83 (0.80–0.86) *p* < .001	0.80 (0.76–0.83) *p* < .001 0.89 (0.85–0.93) *p* < .001 1.00 (0.85–1.18) *p* = .970 0.89 (0.84–0.94) *p* < .001 0.67 (0.63–0.72) *p* < .001 0.86 (0.84–0.90) *p* < .001	1.40 (1.30–1.51) *p* < .001 1.27 (1.18–1.37) *p* < .001 1.18 (0.90–1.56) *p* = .032 1.22 (1.10–1.35) *p* < .001 1.55 (1.38–1.73) *p* < .001 1.19 (1.12–1.27) *p* < .001	1.22 (0.17–1.28) *p* < .001 1.34 (1.28–1.41) *p* < .001 0.90 (0.75–1.08) *p* = .252 1.31 (1.23–1.39) *p* < .001 1.36 (1.26–1.47) *p* < .001 0.99 (0.95–1.03) *p* = .623
Model 3	(Model 2) + Age, gender, LTC status	Asian Black Chinese Mixed Other White-other	0.68 (0.65–0.71) *p* < .001 0.79 (0.75–0.83) *p* < .001 1.10 (0.95–1.29) *p* = .210 0.81 (0.77–0.86) *p* < .001 0.59 (0.55–0.64) *p* < .001 0.84 (0.81–0.87) *p* < .001	0.79 (0.75–0.83) *p* < .001 0.89 (0.85–0.93) *p* < .001 1.00 (0.84–1.18) *p* = .986 0.90 (0.85–0.72) *p* < .001 0.67 (0.62–0.72) *p* < .001 0.87 (0.84–0.90) *p* < .001	1.43 (1.32–1.54) *p* < .001 1.27 (1.17–1.38) *p* < .001 1.22 (0.92–1.63) *p* = .168 1.23 (1.10–1.36) *p* < .001 1.54 (1.37–1.74) *p* < .001 1.17 (1.10–1.25) *p* < .001	1.19 (1.13–1.25) *p* < .001 1.32 (1.26–1.39) *p* < .001 0.83 (0.69–1.00) *p* = .050 1.20 (1.12–1.28) *p* < .001 1.36 (1.25–1.47) *p* < .001 0.97 (0.93–1.01) *p* = .096
Model 4	(Model 3) + Baseline severity PHQ9, Baseline severity GAD7, Baseline severity WSAS, diagnosis/presenting problem, social phobia, agoraphobia, specific phobia	Asian Black Chinese Mixed Other White-other	0.80 (0.77–0.85) *p* < .001 0.96 (0.91–1.00) *p* = .116 1.21 (1.01–0.97) *p* = .037 0.91 (0.86–0.97) *p* = .006 0.72 (0.87–0.94) *p* < .001 0.90 (0.87–0.94) *p* < .001	0.83 (0.79–0.87) *p* < .001 0.98 (0.93–1.04) *p* = .522 1.20 (0.99–1.48) *p* = .070 0.94 (0.88–1.10) *p* = .095 0.70 (0.64–0.76) *p* < .001 0.87 (0.83–0.91) *p* < .001	1.52 (1.40–1.65) *p* < .001 1.19 (1.08–1.30) *p* < .001 0.93 (0.66–1.30) *p* = .662 1.17 (1.03–1.31) *p* = .012 1.68 (1.47–1.92) *p* < .001 1.16 (1.07–1.25) *p* < .001	1.08 (1.03–1.14) *p* = .004 1.21 (1.15–1.28) *p* < .001 0.79 (0.64–0.97) *p* = .023 1.13 (1.05–1.21) *p* = .001 1.23 (1.13–1.35) *p* < .001 0.92 (0.88–0.97) *p* = .001
Model 5	(Model 4) + IMD, employment status, medication status	Asian Black Chinese Mixed Other White-other	0.82 (0.78–0.87) *p* < .001 1.02 (0.97–1.08) *p* = .478 1.20 (1.00–1.44) *p* = .054 0.95 (0.89–1.02) *p* = .155 0.79 (0.72–0.87) *p* < .001 0.93 (0.89–0.97) *p* < .001	0.84 (0.79–0.88) *p* < .001 1.04 (0.99–1.11) *p* = .133 1.20 (0.97–1.47) *p* = .088 0.97 (0.90–1.04) *p* = .437 0.76 (0.70–0.83) *p* < .001 0.89 (0.85–0.93) *p* < .001	1.48 (1.35–1.62) *p* < .001 1.10 (1.00–1.21) *p* = .057 1.02 (0.71–1.46) *p* = .898 1.18 (1.05–1.34) *p* = .008 1.60 (1.38–1.84) *p* < .001 1.18 (1.09–1.28) *p* < .001	1.07 (1.02–1.14) *p* = .008 1.17 (1.11–1.24) *p* < .001 0.82 (0.66–1.01) *p* = .068 1.10 (1.02–1.18) *p* = .013 1.18 (1.08–1.30) *p* < .001 0.92 (0.87–0.96) *p* < .001

When controlling for socio-demographic and clinical factors (Model 5) individuals from Asian (OR = 0.82 [95% CI 0.78–0.87]), ‘Other’ (OR = 0.79 [95% CI 0.72–0.87]) and White-other (0.93 [95% CI 0.89–0.97]) ethnic groups were significantly less likely to reliably recover than the White-British group. They were also less likely to reliably improve: Asian (OR = 0.84 [95% CI 0.79–0.88]); ‘Other’ (OR = 0.76 [95% CI 0.70–0.83]), and more likely to reliably deteriorate, as were those from the Mixed ethnicity group, all relative to the White-British group: Asian (OR = 1.48 [95% CI 1.35–1.62]); Mixed (OR = 1.18 [95% CI 1.05–1.34]); ‘Other’ (OR = 1.60 [95% CI 1.38–1.84]), and White-other (OR = 1.18 [95% CI 1.09–1.28]). All ethnic groups except for the Chinese showed higher odds of drop-out relative to the White-British group.

### Organization-level factor interactions

There was evidence of an interaction between ethnicity and the time between referral and assessment on drop-out, such that individuals in the Asian group were slightly less likely to drop out compared to White-British individuals when the primary waiting time increased: OR = 0.99 [95% CI 0.99–1.00].

There was evidence of an interaction between ethnicity and secondary waiting time, such that an increased waiting time between assessment and treatment was associated with slightly increased odds of deterioration for people in the Mixed group (OR = 1.02 [95%CI 1.00–1.02]) compared to the White-British group.

There evidence of an interaction between ethnicity groups and the number of treatment sessions received. A higher number of sessions was associated with a small increase in odds of reliable recovery for the Black group (OR = 1.01 [95% CI 1.00–1.03]) and for the Mixed group (OR = 1.02 [95% CI 1.00–1.03]) compared to the White-British group.

The odds of dropping out of treatment was lower when the number of sessions was higher for people in the Asian (OR = 0.97 [95% CI 0.94–0.99]) and White-other (OR = 0.97 [95% CI 0.95–0.99]) groups compared to the White-British group.

A higher number of face-to-face sessions was associated with slightly higher odds of reliable recovery for Black (OR = 1.01[95% CI 1.00–1.02]) and Mixed individuals (OR = 1.02 [95% CI 1.01–1.04]) compared to the White-British group. Higher odds of deterioration were seen for people in the Chinese group when the number of face-to-face sessions was higher (OR = 0.1.07 [95% CI 1.00–1.13]) compared to people in the White-British group. Relative to the White-British group, those in the Black ethnicity group had significantly lower odds of drop-out with a higher number of face-to-face sessions: OR = 0.98 [95% CI 0.96–1.00].

A higher number of high intensity (HI) sessions was associated with slightly greater odds of reaching reliable recovery for the Mixed ethnicity group (OR = 1.02 [95% CI 1.01–1.03]), lower odds of reliable improvement in the Chinese ethnicity group (OR = 0.96 [95% CI 0.92–0.099]), and with slightly lower odds of drop-out for the Asian group (OR = 0.98 [95% CI 0.97–1.00]), relative to White-British individuals. No significant interactions were observed for deterioration outcomes.

There was no evidence of interactions between the following organization-level variables and ethnicity for the effects on treatment outcomes: method of access via GP referral, method of access via self-referral, form used to provide treatment—via video-call.

The supplementary material contains further information describing the organization-level variables, how these were operationalized, and the results of the analyses performed.

### Sub-group analyses by gender

Minoritized ethnicity males were more likely to drop out of treatment compared to White-British males (OR = 0.86 [95% CI 0.80–0.91]), however this was not the case for minoritized ethnicity females OR = 1.01[95% CI 0.97–1.06]). Minoritized ethnic males and females had poorer outcomes compared to their White-British counterparts (males, recovery: OR = 1.13 [95% CI 1.07–1.20]; females, recovery: (OR = 1.07 [95% CI 1.03–1.11]; males, improvement: OR = 1.13 [95% CI 1.07–1.20]); females, improvement: OR = 1.08 [95% CI 1.04–1.13],; males, deterioration: OR = 0.72 [95% CI 0.65–0.80]); females, deterioration: OR = 0.84 [95% CI 0.78–0.91]).

When exploring differences by ONS ethnic group, there were also some differences between the two genders. Chinese females experienced higher odds of recovery compared to White-British females, even when controlling for socio-demographic and clinical factors (OR = 1.25 [95% CI 1.02–1.53]), but this was not the case for Chinese males (OR = 1.06 [95% CI 0.72–1.56]). Black males were more likely to drop out of treatment than White-British males (OR = 1.42 [95% CI 1.28–1.58]) but the same was not true for females (OR = 1.08 [95% CI 1.01–1.16]). Females in the White-other group had lower odds of drop-out when compared to White-British females (OR = 0.86 [95% CI 0.82–0.91]), while no such difference observed for White-other males when compared to White-British males (OR = 1.04 [95% CI 0.95–1.13]). A consistent finding across female and male sub-groups was that people in the Asian, ‘Other’ and White-other groups were more likely to experience a reliable deterioration relative to the White-British groups, despite accounting for socio-demographic and clinical factors. Full results from the stratified gender analyses are provided as supplementary material.

## Discussion

Observed (unadjusted) differences in outcomes between White-British individuals and people from different minoritized ethnic groups was reduced and, in some cases, disappeared entirely when accounting for socio-demographic and clinical factors. In whole-sample analyses in this study, people in the Black group were more likely to drop out, but not to experience worse treatment outcomes than White-British individuals when controlling for other factors. People in the Asian, ‘Other’ and White-other groups continued to experience poorer outcomes indicating that further efforts are required to enable people from these communities to benefit equally from talking therapies. Asian, Mixed and ‘Other’ groups also showed higher odds of disengagement. Existing research has identified poorer outcomes for people from Black and Asian communities [37], yet recent NHS reports suggest a decreasing disparity on some outcomes between White and other ethnic groups [8].

Access and outcome discrepancies experienced by Asian people are well-researched [38, 39]. The results from the current study might be understood in terms of identified challenges, such as awareness, cultural differences, stigma and social isolation [40] which might persist. Steps services might take to support at-risk groups could include consistent adoption of recommendations to ensure treatment suitability [41]. The reasons behind observations of poorer outcomes could be explored further using qualitative methods, both with patients belonging to at-risk groups and with clinicians delivering treatment.

A higher number of treatment sessions was associated with better outcomes for Black and Mixed individuals. Similarly, a higher number of sessions was associated with reduced drop-out for Asian and White-other groups. This is reinforced by previous research which has suggested that time waited to start treatment can lead to negative outcomes [11] and the current study found that primary waiting time was associated with increased risk of deterioration for people in the Mixed group. Reducing waiting times and increasing the number of sessions might support improved outcomes for these groups. While both of these actions are likely to be challenging for services to employ given the increasing demand for talking therapies and workforce shortages, innovative use of digital technologies to offer support remotely [42, 43] or to keep patients informed about their wait for treatment [44] are ways that services could address these challenges. Additionally, making organization-level adaptations such as supporting access to treatment in more accessible spaces (such as community, religious and non-healthcare settings) [12] can support people to access care more quickly, which is associated with improved outcomes and with lower likelihood of requiring more intensive and longer treatments [11]. As such, these adaptations might also be used to increase access within existing limited resources.

Despite observing some consistencies in certain outcomes for some ethnic groups across genders (such as for White-other groups and deterioration), there was variation when both gender groups were analysed independently. Previous research has identified that factors such as experiences of discrimination, cultural insensitivity and power imbalances impact access to mental health services for people from minoritized ethnic groups [10]. Research into differences in mental health service use across genders has suggested factors such as gendered societal expectations, mental health literacy and methods of communicating with health professionals can impact engagement [45–47]. This paper highlights the importance of services understanding how gender, including varying cultural perceptions of gender, may influence outcomes for different ethnic groups. Factors impacting treatment are likely to vary significantly between cultures and age groups and as such, future research involving more focussed exploration of specific factors that lead people to disengage from treatment could help services to understand what could reduce drop-out.

Factors associated with gender, may interact with organization-level factors to influence outcomes differently between ethnic groups. Existing research suggests gender inequalities in engagement are associated with intersecting factors of ethnicity, religion and socio-economic status [20]. Organization-level variables appeared to interact with ethnicity to influence outcomes in some cases, but the presence of a significant interaction did not necessarily result in significant outcome disparities when comparing people from minoritized groups to White-British people. These findings might be understood in terms of the intersecting gender and ethnicity characteristics that may be differentially impacted by societal or cultural factors contributing to differences in outcomes [20, 48].

The study included a binary categorisation of ethnic groups, and analyses using ONS ethnicity categories. Amalgamating people from minoritized ethnic groups into one group was useful to explore comparisons in terms of outcomes achieved by people who are racialised or perceived as ‘*non*-*White*’ versus those who are ‘*White-British’*; this approach reflects how people from minoritized communities can be socially assigned as ‘minorities’ and report differential treatment within services and healthcare discrimination [3, 5]. Across gender sub-group analyses, the White-other group experienced higher odds of deterioration compared to White-British individuals. This is interesting when considering the impact of ethnicity and discrimination on outcomes, especially in light of the discussion around social assignment of minority status. The White-other ethnic category includes people of European descent, including immigrants. A speculative explanation might be that these groups are racialised as ‘White’ by society, yet factors such as recency of immigration or not having English as their first language might counteract the beneficial effects of treatment. Despite being socialised as ‘minorities’, people from Black, Asian or other groups may experience a protective factor in comparison to people in the White-other category, if they were born in the UK or have been residents for a longer period of time. There is evidence to suggest that recency of immigration can impact psychological treatment outcomes [49]. This might be considered in further studies exploring differences in outcomes between ethnic groups, especially when making decisions about grouping by ethnicity. The study highlights the risk of ‘hiding’ potential differences between discrete groups which could lead to missed opportunities to improve outcomes for certain at-risk populations.

### Limitations

Using ONS ethnicity categories allowed for more granular exploration of differences between groups, yet categories remain high-level. The way in which variables such as ethnicity are structured when they are collected routinely for the purposes of clinical practice limited the number categories available for analysis as they were based on census data, and there was measurement error introduced by the cross-over of constructs related to race, ethnicity and nationality, all captured in the single variable. Amalgamating discrete ethnic groups for analysis risks erasure of important differences and nuances between groups and is an extant limitation of inequalities research; the risk of ‘hiding’ inequalities by grouping people together may result in misleading conclusions. Additionally, the data did not allow for analysis of impact of immigration or refugee/asylum seeker status on outcomes.

Categories of ‘male’ and ‘female’ are the only options patients had to self-identify their gender at the time data used in this study were collected. These are terms which more accurately refer to biological sex rather than gender, and their use limits the exploration of potential intersecting outcomes for people who identify their gender outside of these two categories.

Which factors should be adjusted for as confounders is contentious given the lack of consensus on causal pathways and the impact of decisions about adjustments on the interpretation of findings. Further research, using an updated sample of data would be of use to confirm the associations found in this study.

There was a large number of tests conducted for this study which might have increased the chance of making Type 1 errors [50]. However, in line with recommendations by Rothman (1990) [51] and Perneger (1998) [52] no adjustments were made to mitigate for this. All analyses have been presented irrespective of statistical significance and thresholds for statistical significance were not used to inform interpretations of the findings. This does not remove the possibility of some Type 1 errors but does prevent many of the issues related to data-mining or ‘*p*-hacking’ [50, 53].

### Implications

The results show ethnic and gender differences in outcomes. Controlling for other factors did not reduce the likelihood of treatment drop-out for Black, Asian or ‘Other’ individuals, suggesting challenges in treatment retention and engagement remain. People in the Asian, ‘Other’ and White-other groups experienced worse outcomes than White-British people across all outcomes, suggesting that additional changes to treatment may be necessary to improve outcomes. The results provide insight into the different organization-level factors that might be adapted as part of IAPT care to improve outcomes for people with different characteristics. Interactions observed regarding drop-out for the Asian group (for factors including number of sessions received and primary waiting time) suggest these organization-level factors may play an important role in treatment retention. Increasing the number of sessions offered and reducing waiting times are actions that services could adopt and monitor the impact on outcomes. Further research is needed to better understand which other factors may interact with ethnicity to influence outcomes in Asian, ‘Other’ and White- other groups. Ethnic group might not be the driving factor for these differences which may be attributed to interactions between organization-level factors and other variables (perhaps linked to ethnicity) not explored in this study. Existing research shows that making adaptations to organization-level factors can improve outcomes for people from minoritized ethnic groups, but the results of this study indicate that it is likely that a variety of factors contribute to the success or failure of treatment to lead to better outcomes for different people. Finally, consideration should be given to ethnic group categorisation, due to the potential for issues impacting discrete groups to be missed. Future research should avoid amalgamating all ‘White’ groups as this may lead to failure to identify hidden inequalities which may be linked to immigration and other factors.

### Supplementary Information

Below is the link to the electronic supplementary material.

Supplementary file1 (DOCX 61 KB)

Supplementary file2 (DOCX 2339 KB)

## Data Availability

There are restrictions on the availability of data used in this study in accordance with the NCEL agreement (project reference: 00519-IAPT). Data were used under license for the current study, and so are not publicly available.

## Code availability

STATA code is available upon request to the corresponding author.

## Material availability

Relevant research material associated with this manuscript is provided as supplementary material.
