# Supplementary material for: Differences in psychological treatment outcomes by ethnicity and gender: an analysis of individual patient data
Source: Soc Psychiatry Psychiatr Epidemiol. 2024 Feb 6;59(9):1519–31. doi: 10.1007/s00127-024-02610-8 (PMC11343885; doi:10.1007/s00127-024-02610-8)
Supplement: Supplementary file 1 — Supplementary file1 (DOCX 61 KB) [file 127_2024_2610_MOESM1_ESM.docx]

Differences in psychological treatment outcomes by ethnicity and gender: an analysis of individual patient data

Laura-Louise C. Arundell*^;^ Rob Saunders; Joshua E. J. Buckman; Glyn Lewis; Joshua Stott; Satwant Singh; Renuka Jena; Syed Ali Naqvi; Judy Leibowitz; Stephen Pilling.

*[l.arundell@ucl.ac.uk](mailto:l.arundell@ucl.ac.uk); CORE Data Lab, Centre for Outcomes Research and Effectiveness, Research Department of Clinical, Educational and Health Psychology, University College London, Gower Street, London, UK.

SUPPLEMENTARY MATERIAL 1

Figure S1

[LEGEND] Figure S1: data exclusion flowchart

Remove if missing ‘End of treatment date’

n= 14,657

n = 483,683

Remove if <2 treatment sessions (n = 304,055) OR incomplete outcomes data (n= 7,275)

n= 311,330

n = 469,026

n = 157,696

Remove not at ‘caseness’

n=17,078

n = 140,618

Remove duplicates

n= 17,498

Remove if age<18 years

n = 711

n = 123,120

Remove missing age

n = 3

n = 122,409

n = 122,406

Remove missing ethnicity code

n = 7,957

n = 114,449

Remove missing IMD decile

n = 1,730

Remove missing LTC status

n = 14, 656

n = 112,719

Remove gender not stated, gender missing and gender not known

n= 255

Remove males

n= 37,209

**Analytic data sample**

**n = 98,063**

n = 97,808

Analytic data subsample of MALES with complete data

**n = 31,515**

Analytic data subsample of FEMALES with complete data

**n = 66,293**
