# Supplementary material for: Differences in psychological treatment outcomes by ethnicity and gender: an analysis of individual patient data
Source: Soc Psychiatry Psychiatr Epidemiol. 2024 Feb 6;59(9):1519–31. doi: 10.1007/s00127-024-02610-8 (PMC11343885; doi:10.1007/s00127-024-02610-8)
Supplement: Supplementary file 2 — Supplementary file2 (DOCX 2339 KB) [file 127_2024_2610_MOESM2_ESM.docx]

Differences in psychological treatment outcomes by ethnicity and gender: an analysis of individual patient data

Laura-Louise C. Arundell*^;^ Rob Saunders; Joshua E. J. Buckman; Glyn Lewis; Joshua Stott; Satwant Singh; Renuka Jena; Syed Ali Naqvi; Judy Leibowitz; Stephen Pilling.

*[l.arundell@ucl.ac.uk](mailto:l.arundell@ucl.ac.uk); CORE Data Lab, Centre for Outcomes Research and Effectiveness, Research Department of Clinical, Educational and Health Psychology, University College London, Gower Street, London, UK.

SUPPLEMENTARY MATERIAL 2

Appendices

**Contents**

Appendix 1: Statistical tests for research question 1

Appendix 2: Statistical tests for research question 2

Appendix 3: Statistical tests for research question 3

Appendix 4: Results – whole-sample logistic regression models with interactions

Appendix 5: Results – female sub-group analyses

Appendix 6: Results – male sub-group analyses

# Appendix 1: Statistical tests for research question 1

***Differences in unadjusted outcomes different between people from minoritized ethnicity groups, compared to White-British individuals***

Table 1: Analyses performed for research question 1

| **RQ1:**  **Are unadjusted outcomes different between people from minoritized ethnicity groups, compared to White British individuals?** | | |
| --- | --- | --- |
| **Measure(s)** | **Data** | **Method(s)** |
| Reliable recovery | Categorical (recovered =1, not recovered =0) | Compare outcomes between ethnic groups using chi-square test  Compare outcomes between ethnic groups in subsample of **males** using chi-square test  Compare outcomes between ethnic groups in sub-sample of **females** using chi-square test |
| Change score PHQ-9 | Continuous (0-27) | Compare means from white/minoritized ethnicity (binary) people using independent samples t-test  Compare means from white/minoritized ethnicity (binary) **males** using independent samples t-test  Compare means from white/minoritized ethnicity (binary) **females** using independent samples t-test |
| Change score GAD-7 | Continuous (0-21) | Compare means from white/minoritized ethnicity (binary) people using independent samples t-test  Compare means from white/minoritized ethnicity (binary) **males** using independent samples t-test  Compare means from white/minoritized ethnicity (binary) **females** using independent samples t-test |
| Attrition/drop-out | Categorical (drop-out = 1, no drop-out = 0) | Compare outcomes between ethnic groups using chi-square test  Compare outcomes between ethnic groups - **males** using chi-square test  Compare outcomes between ethnic groups - **females** using chi-square test |
| Deterioration | Categorical (deteriorated =1, not deteriorated = 0) | Compare outcomes between ethnic groups using chi-square test  Compare outcomes between ethnic groups in subsample of **males** using chi-square test  Compare outcomes between ethnic groups in sub-sample of **females** using chi-square test |
| *Each set of analyses was run on the whole data sample, and then on each sub-sample of males and females.* | | |

# Appendix 2: Statistical tests for research questions 2 and 2.1

***Differences in treatment outcomes between people from minoritized ethnic groups and White-British individuals, independent of particular patient characteristics and factors***

- ***Differences in treatment outcomes between minoritized ethnic females (using Office for National Statistics ethnicity categories) and White-British females, independent of particular patient characteristics and factors***

**Table 2** outlines the regression models used to explore whether ethnic group is associated with outcomes, whilst controlling for several confounders. Each set of analyses was run on the whole data sample, and then on each sub-sample of males and females. Analyses for research question 2 (RQ2) compared outcomes between two binary categories of ethnicity (i.e., a White-British and an aggregate minority ethnic group). Analyses for RQ21 compared outcomes from sub-groups of people from minoritized ethnic groups (i.e., people reported as anything other than White-British according to ONS categories of ethnic group) to outcomes from White-British people using ‘White-British’ as the reference category.

Table 2: Analyses performed for research question 2

| 1. **RQ2:**  **Differences in treatment outcomes between people from minoritized ethnic groups and White-British individuals, independent of particular patient characteristics and factors**   **RQ 2.1:**  **Differences in treatment outcomes between minoritized ethnic females (using Office for National Statistics ethnicity categories) and White-British females, independent of particular patient characteristics and factors** | | | |
| --- | --- | --- | --- |
| **Measure** | **Data** | **Method** | **Models and variables** |
| Reliable recovery | Categorical  (recovered =1, not recovered =0) | Logistic Regression | Model 1: Ethnicity  Model 2: (Model 1) + Service  Model 3: (Model 2) + Age, gender (*for whole sample analyses only*), LTC status  Model 4: (Model 3) + Baseline severity PHQ9, Baseline severity GAD7, Baseline severity WSAS, diagnosis/presenting problem, social phobia, agoraphobia, specific phobia  Model 5: (Model 4) + IMD, employment status, Medication status |
| Reliable improvement | Categorical  (improvement =1, no improvement =0) | Logistic Regression | Model 1: Ethnicity  Model 2: (Model 1) + Service  Model 3: (Model 2) + Age, gender (*for whole sample analyses only*), LTC status  Model 4: (Model 3) + Baseline severity PHQ9, Baseline severity GAD7, Baseline severity WSAS, diagnosis/presenting problem, social phobia, agoraphobia, specific phobia  Model 5: (Model 4) + IMD, employment status, Medication status |
| Deterioration | Categorical  (deteriorated=1, no deterioration =0) | Logistic Regression | Model 1: Ethnicity  Model 2: (Model 1) + Service  Model 3: (Model 2) + Age, gender (*for whole sample analyses only*), LTC status  Model 4: (Model 3) + Baseline severity PHQ9, Baseline severity GAD7, Baseline severity WSAS, diagnosis/presenting problem, social phobia, agoraphobia, specific phobia  Model 5: (Model 4) + IMD, employment status, Medication status |
| Drop-out | Categorical  (drop-out =1, no drop-out =0) | Logistic Regression | Model 1: Ethnicity  Model 2: (Model 1) + Service  Model 3: (Model 2) + Age, gender (*for whole sample analyses only*), LTC status  Model 4: (Model 3) + Baseline severity PHQ9, Baseline severity GAD7, Baseline severity WSAS, diagnosis/presenting problem, social phobia, agoraphobia, specific phobia  Model 5: (Model 4) + IMD, employment status, Medication status |

# Appendix 3: Statistical tests for research question 3

***Organization-level factors contributing to outcomes for different ethnic groups***

The following regression analysis with an interaction term was performed for each of the outcome measures:

*[****Outcome measure****] =* ***ethnicity*** *x [****organization-level variable****] + service + age, gender (for whole sample analyses only), LTC status + baseline severity PHQ-9, baseline severity GAD-7, baseline severity WSAS, presenting problem, composite phobia + IMD, employment status, psychotropic medication status*

Table 3: Analyses for research question 3 – regression models with interaction terms

| **RQ3: Organization-level factors contributing to outcomes for different ethnic groups** | | | |
| --- | --- | --- | --- |
| **Outcome measures*** | **Data** | **Variable** | **Logistic regression analysis with interaction term** |
| Reliable recovery  Reliable improvement  Deterioration  Attrition/drop out | Categorical  (recovered =1, not recovered =0)  Categorical  (improvement =1, no improvement =0)  Categorical  (deteriorated=1, no deterioration =0)  Categorical  (drop-out =1, no drop-out =0) | Number of sessions | [Outcome measure] = Ethnicity x Number of Sessions + Service + Age, gender (*for whole sample analyses only*), LTC status + Baseline severity PHQ9, Baseline severity GAD7, Baseline severity WSAS, diagnosis/presenting problem, composite phobia + IMD, employment status, Medication status |
| Reliable recovery  Reliable improvement  Deterioration  Attrition/drop out | Categorical  (recovered =1, not recovered =0)  Categorical  (improvement =1, no improvement =0)  Categorical  (deteriorated=1, no deterioration =0)  Categorical  (drop-out =1, no drop-out =0) | Method of access/Referral Source (dummy coded) | [Outcome measure] = Ethnicity x Referral source + Service + Age, gender (*for whole sample analyses only*), LTC status + Baseline severity PHQ9, Baseline severity GAD7, Baseline severity WSAS, diagnosis/presenting problem, composite phobia + IMD, employment status, Medication status |
| Reliable recovery  Reliable improvement  Deterioration  Attrition/drop out | Categorical  (recovered =1, not recovered =0)  Categorical  (improvement =1, no improvement =0)  Categorical  (deteriorated=1, no deterioration =0)  Categorical  (drop-out =1, no drop-out =0) | Form used to provide treatment (face-to-face, telephone/video) | [Outcome measure] = Ethnicity x Form used to provide treatment + Service + Age, gender (*for whole sample analyses only*), LTC status + Baseline severity PHQ9, Baseline severity GAD7, Baseline severity WSAS, diagnosis/presenting problem, composite phobia + IMD, employment status, Medication status |
| Reliable recovery  Reliable improvement  Deterioration  Attrition/drop out | Categorical  (recovered =1, not recovered =0)  Categorical  (improvement =1, no improvement =0)  Categorical  (deteriorated=1, no deterioration =0)  Categorical  (drop-out =1, no drop-out =0) | Time waited to assessment (number of days from referral to assessment) | [Outcome measure] = Ethnicity x Time waited to assessment + Service + Age, gender (*for whole sample analyses only*), LTC status + Baseline severity PHQ9, Baseline severity GAD7, Baseline severity WSAS, diagnosis/presenting problem, composite phobia + IMD, employment status, Medication status |
| Reliable recovery  Reliable improvement  Deterioration  Attrition/drop out | Categorical  (recovered =1, not recovered =0)  Categorical  (improvement =1, no improvement =0)  Categorical  (deteriorated=1, no deterioration =0)  Categorical  (drop-out =1, no drop-out =0) | Time waited to begin treatment (number of days assessment to treatment/secondary wait) | [Outcome measure] = Ethnicity x Time waited to treatment + Service + Age, gender (*for whole sample analyses only*), LTC status + Baseline severity PHQ9, Baseline severity GAD7, Baseline severity WSAS, diagnosis/presenting problem, composite phobia + IMD, employment status, Medication status |
| Reliable recovery  Reliable improvement  Deterioration  Attrition/drop out | Categorical  (recovered =1, not recovered =0)  Categorical  (improvement =1, no improvement =0)  Categorical  (deteriorated=1, no deterioration =0)  Categorical  (drop-out =1, no drop-out =0) | Treatment intensity (number of high intensity treatment sessions) | [Outcome measure] = Ethnicity x Treatment intensity + Service + Age, gender (*for whole sample analyses only*), LTC status + Baseline severity PHQ9, Baseline severity GAD7, Baseline severity WSAS, diagnosis/presenting problem, composite phobia + IMD, employment status, Medication status |

A likelihood ratio test was used to explore whether the inclusion of an interactions term improved adjusted models (model 5).

# Appendix 4: Results – whole-sample logistic regression models with interactions

Table 4: Results from the likelihood-ratio test used to compare the adjusted model (model 5) with the inclusion of an interaction term

| **Organization-level variable** | **Reliable recovery** | **Reliable improvement** | **Deterioration** | **Dropout** |
| --- | --- | --- | --- | --- |
| Number of sessions | *X^2^* (6) = 26.71, *p<*.001) | *X^2^* (6) = 9.10, *p*=.168 | *X^2^* (6) = 7.42, *p*=.283 | *X^2^* (6) = 15.72, *p*=.015 |
| Referral source (GP/self-referral/other) | *X^2^* (12) = 16.81, *p*=.156) | Omitted* | Omitted* | *X^2^* (12) = 22.78, *p*=.030 |
| Number of face-to-face sessions | *X^2^* (6) = 24.16, *p*<.001) | *X^2^* (6) = 9.22, *p*=.162 | *X^2^* (6) = 8.11, *p*=.230 | *X^2^* (6) = 9.98 *p*=.125 |
| Number of telephone sessions | *X^2^* (6) = 1.62, *p*<.001) | *X^2^* (6) = 2.45, *p*=.874 | *X^2^* (6) = 7.72, *p*=.256 | *X^2^* (6) = 3.46 *p*=.749 |
| Number of video call sessions | Omitted* | Omitted* | Omitted* | Omitted* |
| Number of days from referral to assessment (primary wait) | *X^2^* (6) = 9.38, *p*=.153) | *X^2^* (6) = 4.64 *p*=.590 | *X^2^* (6) = 3.00, *p*=.809 | *X^2^* (6) = 13.58 *p*=.034 |
| Number of days assessment to treatment (secondary wait) | *X^2^* (6) = 2.59, *p*=.856) | *X^2^* (6) = 2.35, *p*=.884 | *X^2^* (6) = 7.50, *p*=.277 | *X^2^* (6) = 7.48, *p*=.278 |
| Number of high intensity treatment sessions | *X^2^* (6) = 16.23, *p*=.012) | *X^2^* (6) = 7.22, *p*=.301 | *X^2^* (6) = 6.40, *p*=.380 | *X^2^* (6) = 7.00 *p*=.321 |
| **Missing values led to unequal observations between models, restricting comparison using the likelihood-ratio test.* | | | | |

Inclusion of interaction terms resulted in improvement in several of the models (**Table 4**). The results from the improved models (i.e., *p*<.005) are provided below.

***Organization-level factors contributing to outcomes for different ethnic groups***

## **Reliable recovery**

Table 5: Results from logistic regression models with interactions for the whole analytic data sample – reliable recovery outcome. Included are the factors for each ethnic group that resulted in odds ratios with a p-value of <.05. (reference category = White-British)

| **Reliable recovery** | | |
| --- | --- | --- |
| **Organization-level variables** | **Ethnic group** | |
|  | **Black** | **Mixed** |
| Number of sessions | OR=1.01 (CI: 1.00-1.03),  *p=.*015 | OR=1.02 (CI:1.00-1.03),  *p=.*003 |
| Form used to provide treatment (number of face-to-face sessions) | OR=1.01 (CI:1.00-1.02), *p=.*012 | OR=1.02 (CI:1.00-1.04), *p=.*002 |
| Number of high intensity treatment sessions | -- | OR=1.02 (CI:1.01-1.03), *p=.*004 |


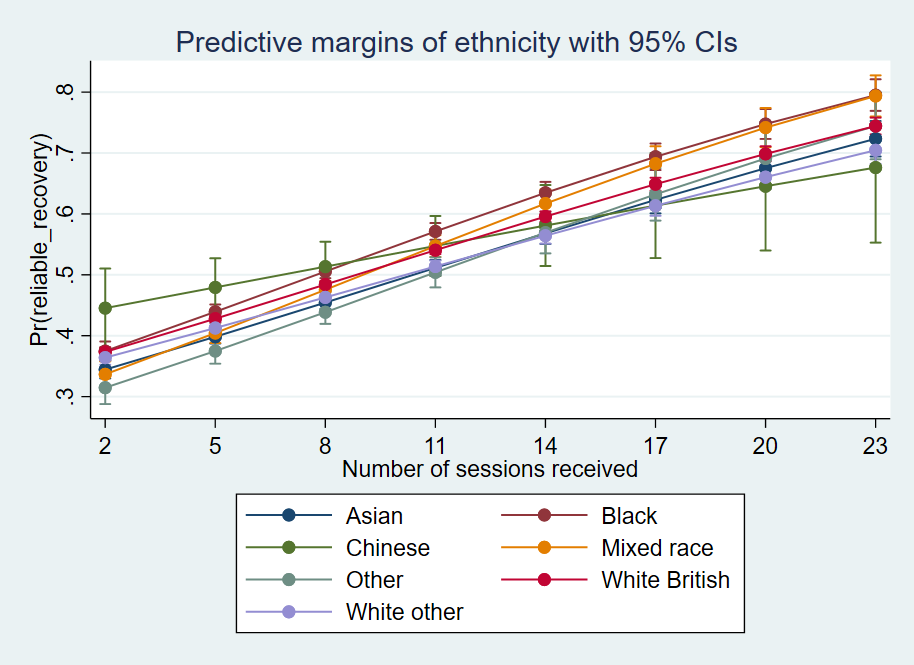


Figure 1: Graph to show ethnic group and number of treatment sessions received as predictors of reliable recovery (whole sample)


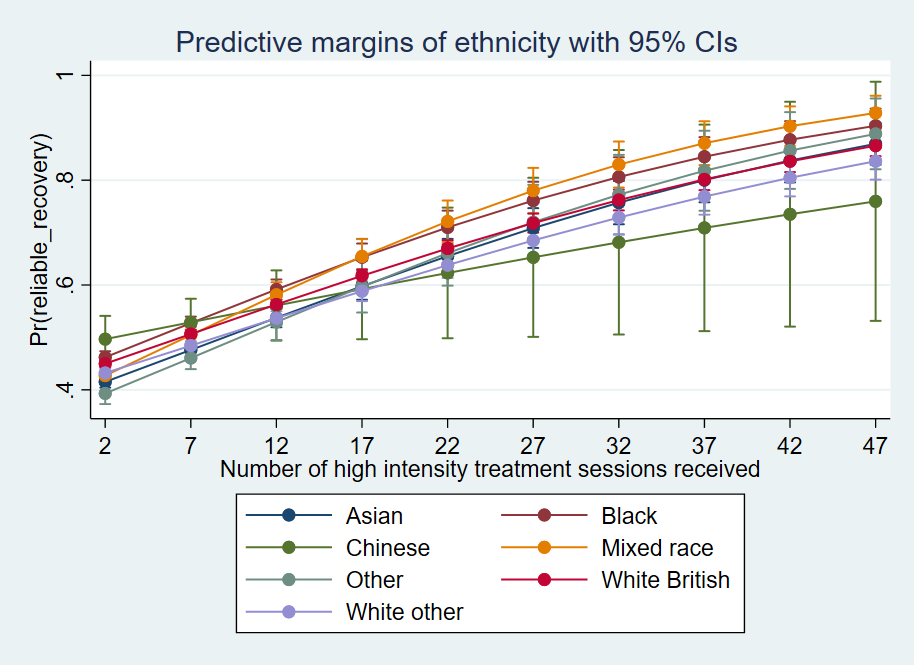


Figure 2: Graph to show ethnic group and number of high intensity treatment sessions received as predictors of reliable recovery (whole sample)


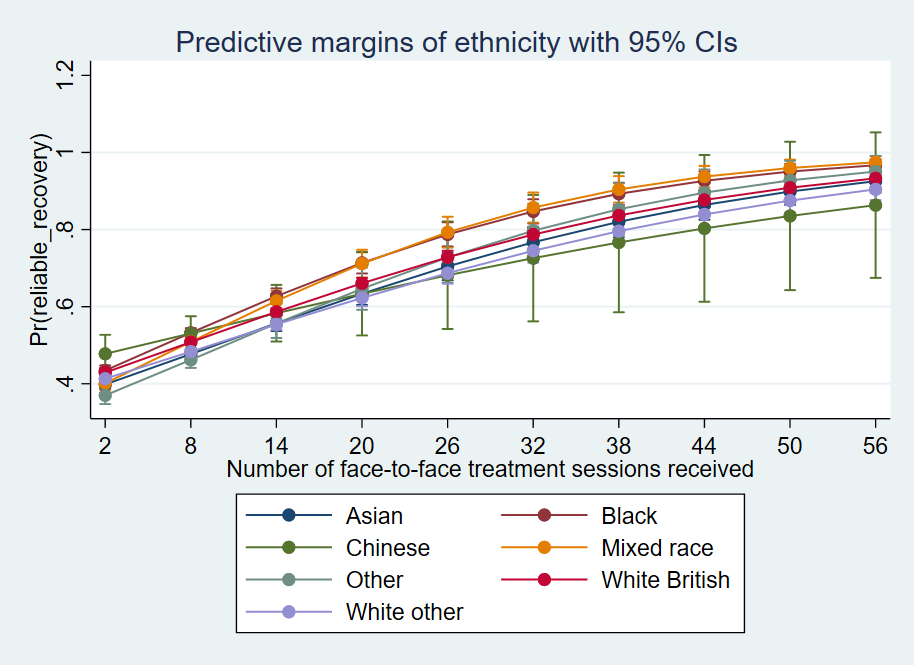


Figure 3: Graph to show ethnic group and number of face-to-face treatment sessions received as predictors of reliable recovery (whole sample)

## **Reliable improvement**

Table 6: RQ3 results for the whole analytic data sample – reliable improvement outcome. Included are the factors for each ethnic group that resulted in odds ratios with a p-value of <.05. (reference category = White-British)

| **Reliable improvement** | |
| --- | --- |
| **Organization-level variables** | **Ethnic group** |
|  | **Chinese** |
| Number of high intensity treatment sessions | OR=0.96 (CI:0.92-0.99), *p*=.025 |


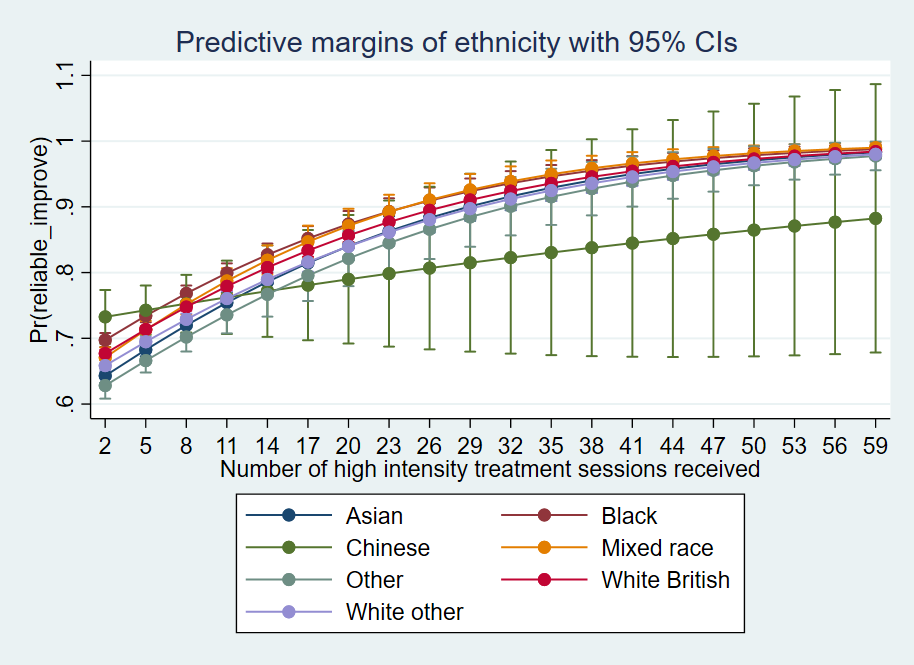


Figure 4: Graph to show ethnic group and number of high intensity treatment sessions received as predictors of reliable improvement (whole sample)

## **Deterioration**

Table 7: RQ3 results for the whole analytic data sample – deterioration outcome. Included are the factors for each ethnic group that resulted in odds ratios with a p-value of <.05. (reference category = White-British)

| **Deterioration** | | |
| --- | --- | --- |
| **Organization-level variables** | **Ethnic group** | |
|  | **Chinese** | **Mixed** |
| Assessment to treatment wait time | -- | OR=1.02 (CI:1.00-1.03), *p=.*013 |
| Form used to provide treatment (number of face-to-face sessions) | OR=1.07 (CI:1.00-1.13), *p=.*041 | -- |
| Form used to provide treatment (number of telephone sessions) | -- | OR=1.07 (CI:1.00-1.15), *p=.*039 |


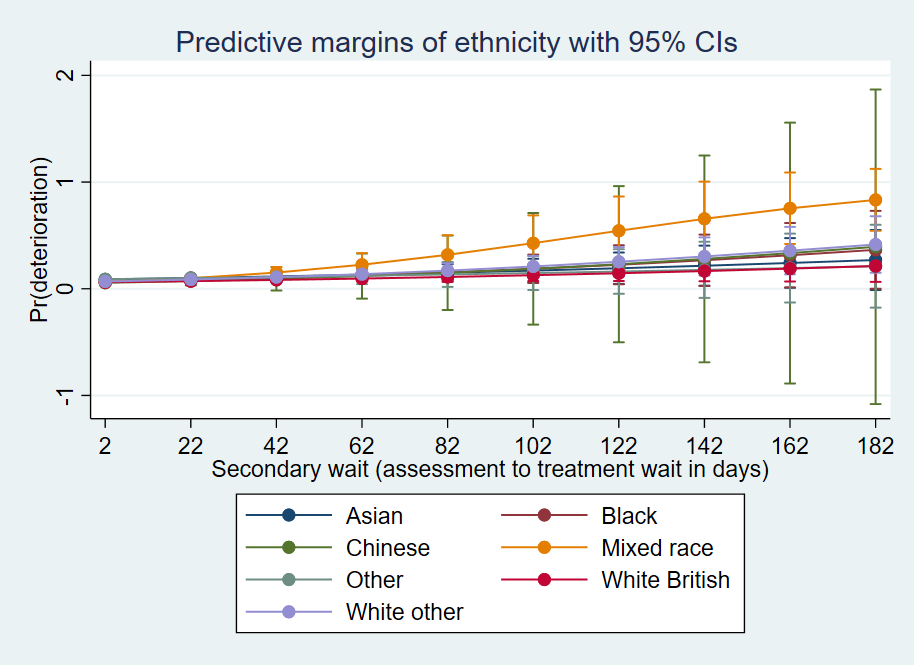


Figure 5: Graph to show ethnic group and secondary wait as predictors of deterioration (whole sample)


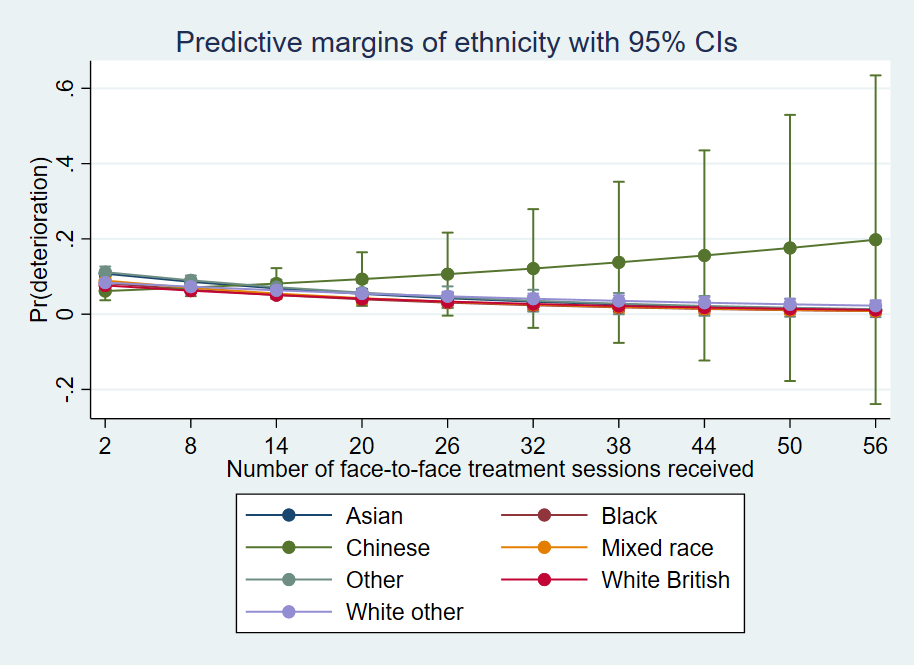


Figure 6: Graph to show ethnic group and number of face-to-face treatment sessions as predictors of deterioration (whole sample)


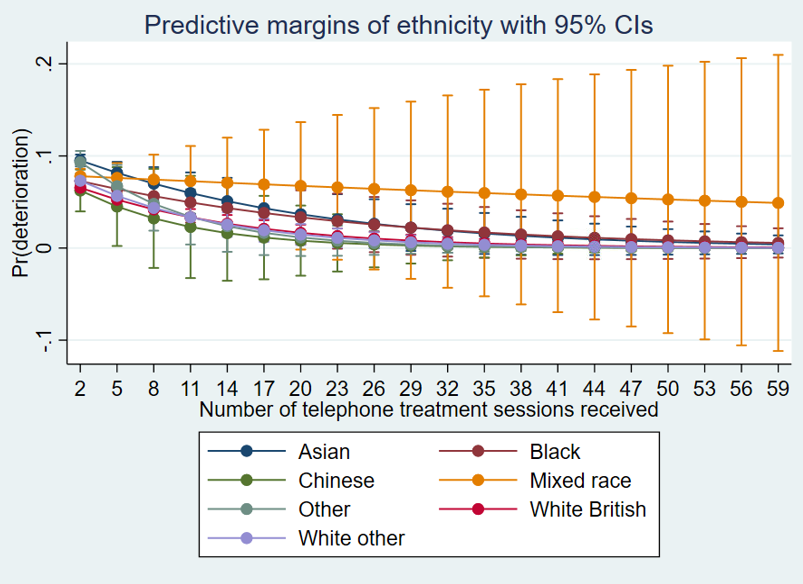


Figure 7: Graph to show ethnic group and number of telephone treatment sessions received as predictors of deterioration (whole sample)

## **Drop-out**

Table 8: RQ3 results for the whole analytic data sample – drop-out. Included are the factors for each ethnic group that resulted in odds ratios with a p-value of <.05. (reference category = White-British)

| **Drop-out** | | | |
| --- | --- | --- | --- |
| **Organization-level variables** | **Ethnic group** | | |
|  | **Asian** | **Black** | **White other** |
| Number of sessions | OR=0.97 (CI:0.94-0.99), *p=.*003 | -- | OR=0.97 (CI:0.95-0.99), *p=.*005 |
| Referral to assessment wait time | OR=0.99 (CI: 0.99-1.00), *p=.*004 | -- | -- |
| Form used to provide treatment (number of face-to-face sessions) | -- | OR=0.98 (CI: 0.96-1.00), *p*=.029 | -- |
| Number of high intensity treatment sessions | OR=0.98 (CI:0.97-1.00), *p=.*024 | -- | -- |


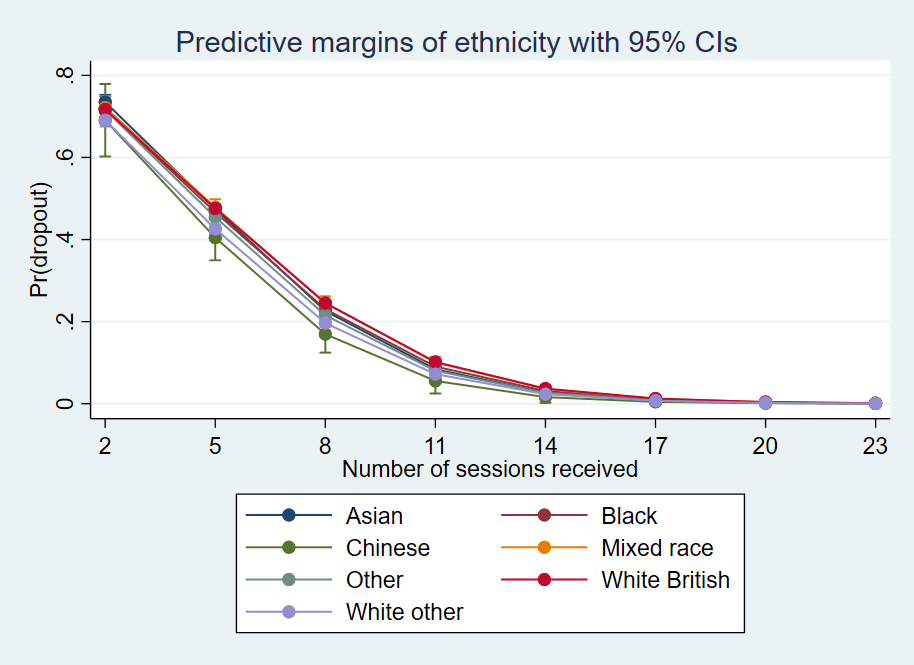


Figure 8: Graph to show ethnic group and number of treatment sessions received as predictors of drop-out (whole sample)


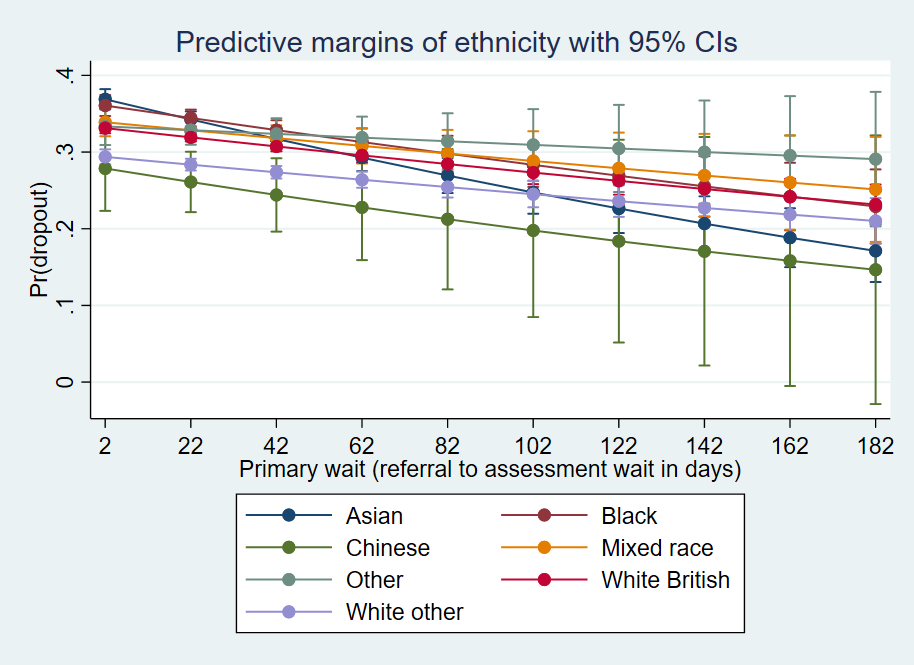


Figure 9: Graph to show ethnic group and primary wait as predictors of drop-out (whole sample)


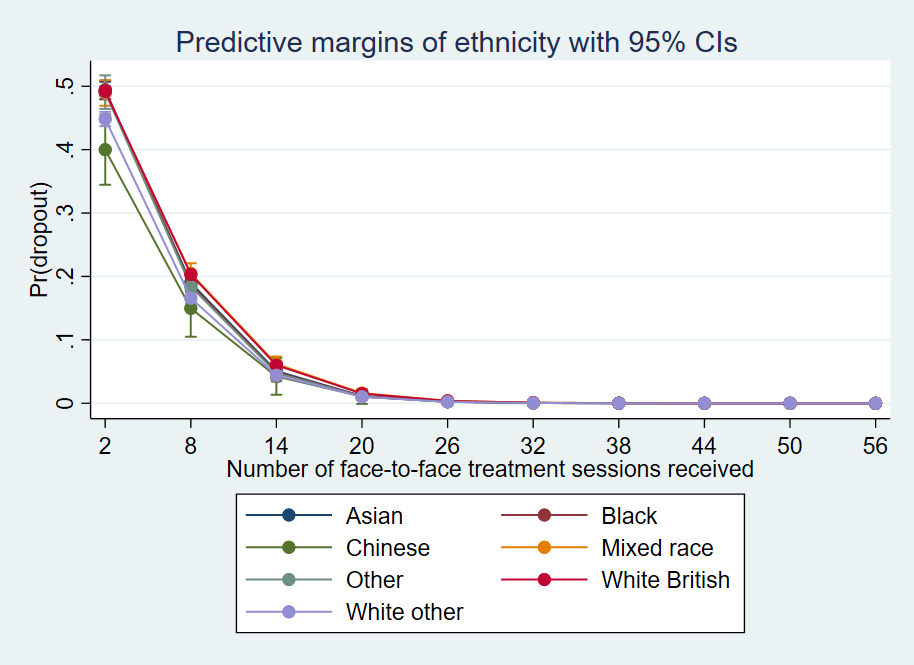


Figure 10: Graph to show ethnic group and number of face-to-face treatment sessions received as predictors of drop-out (whole sample)


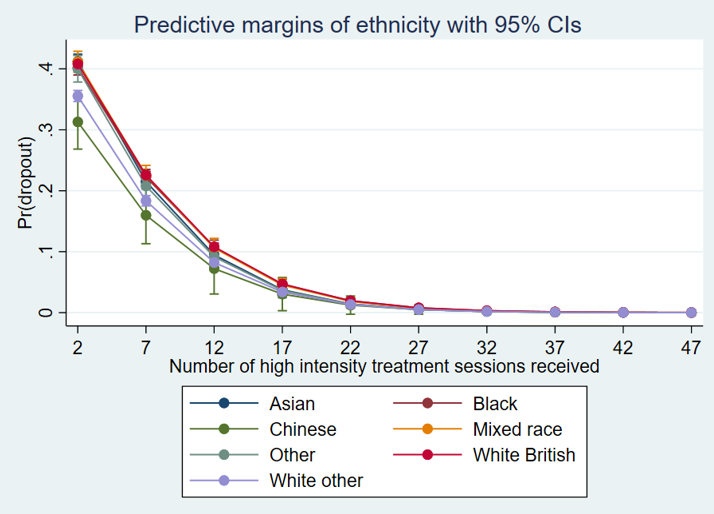


Figure 11: Graph to show ethnic group and number of high intensity treatment sessions received as predictors of drop-out (whole sample)

# Appendix 5: Results - female sub-group analyses

## **Comparison of baseline demographic and clinical characteristics between White-British and Minoritized Ethnicity females**

The subsample of females comprised 66,293 individuals. The ethnicity group breakdown can be seen in **Table 9.** The largest category was White-British (44%) followed by those of Mixed ethnicity (23%). The smallest group was of Chinese individuals who made up less than 1% of the sample.

Table 9: Ethnicity breakdown - female subsample

| **Ethnicity ONS** | **N** | **%** |
| --- | --- | --- |
| White British | 29163 | 43.99 |
| Asian | 7293 | 11 |
| Black | 7610 | 11.48 |
| Chinese | 539 | 0.81 |
| Mixed | 4137 | 23.29 |
| Other | 2494 | 3.76 |
| White - other | 15057 | 22.71 |
| Total | 66923 | |

Several demographic and clinical characteristics between two ethnic groups coded as White-British and an amalgamated group comprising females from minoritized ethnicity groups (i.e., non-White-British), were compared (**Table 10**). Comparison of continuous variables was performed using t-tests, chi-square tests were used for categorical variables.

Table 10: Comparison of baseline demographic and clinical information (continuous and categorical variables) between ethnic groups (female sub-sample)

| **Continuous variables** | **White-British** | | | **Minoritized ethnicity** | | | **Difference** |
| --- | --- | --- | --- | --- | --- | --- | --- |
|  | **N** | **Mean** | **Sd** | **N** | **Mean** | **Sd** | ***p*-value** |
| PHQ-9 | 29161 | 14.76 | 5.58 | 37129 | 15.88 | 5.52 | <.001 |
| GAD-7 | 29161 | 13.91 | 4.35 | 37118 | 14.38 | 4.35 | <.001 |
| WSAS 1 | 24163 | 5.34 | 2.92 | 31853 | 5.69 | 5.66 | <.001 |
| WSAS 2 | 24175 | 3.46 | 1.38 | 31861 | 3.84 | 2.46 | <.001 |
| WSAS 3 | 24174 | 4.29 | 2.38 | 31854 | 4.59 | 2.50 | <.001 |
| WSAS 4 | 24172 | 3.53 | 2.53 | 31849 | 4.02 | 2.63 | <.001 |
| WSAS 5 | 24173 | 3.86 | 2.43 | 31849 | 4.20 | 2.50 | <.001 |
| Social phobia | 28749 | 2.96 | 2.50 | 36234 | 3.38 | 1.66 | <.001 |
| Specific phobia | 28741 | 2.22 | 2.71 | 36213 | 2.65 | 2.88 | <.001 |
| Agoraphobia | 28743 | 2.68 | 2.70 | 36218 | 2.97 | 2.81 | <.001 |
| Number of LI sessions | 29163 | 2.88 | 2.79 | 37130 | 2.96 | 2.81 | <.001 |
| Number of HI sessions | 29163 | 4.85 | 5.63 | 31130 | 4.47 | 5.38 | <.001 |
| Weeks waited from referral to assessment | 29150 | 3.66 | 4.43 | 37114 | 3.94 | 4.75 | <.001 |
| Weeks waited from assessment to treatment | 27215 | 8.63 | 8.34 | 34617 | 9.00 | 8.60 | <.001 |
| Age | 29163 | 38.25 | 14.85 | 37130 | 36.69 | 12.43 | <.001 |
| **Categorical variables** | | **White-British** | | **All minoritized ethnic groups** | | **p-value** | |
|  |  | **N** | % | N | % |  |  |
| Employment | Employed | 23094 | 80.16 | 26780 | 73.14 | <.001 | |
|  | Unemployed | 5716 | 19.84 | 9833 | 26.86 |  |  |
| Existing long-term condition | Yes | 7581 | 27.46 | 9651 | 27.84 | .290 | |
|  | No | 20029 | 72.54 | 25016 | 72.16 |  |  |
| IMD decile | Low | 15529 | 84.57 | 21802 | 90.33 | <.001 | |
|  | High | 2834 | 15.43 | 2334 | 9.67 |  |  |
| Diagnosis/presenting problem | Depression | 10081 | 35.49 | 15263 | 42.25 | <.001 | |
|  | Anxiety disorder | 4501 | 15.84 | 4757 | 13.43 |  |  |
|  | MAAD | 1772 | 6.24 | 2453 | 6.79 |  |  |
|  | PTSD | 541 | 0.19 | 1222 | 3.38 |  |  |
| *GAD-7 = generalised anxiety disorder scale (anxiety symptom measure)*  *HI = high intensity treatment*  *IMD = indices of multiple deprivation*  *LI = low intensity treatment*  *MADD = mixed anxiety and depression*  *PHQ-9 = patient health questionnaire (depression symptom measure)*  *PTSD = post-traumatic stress disorder*  *WSAS = work and social adjustment scale (items 1-5)* | | | | | | | |

## **Differences in unadjusted outcomes between females from minoritized ethnicity groups and White British females**

Differences in a variety of treatment outcomes between an amalgamated group of females from minoritized ethnic backgrounds compared to White-British females were explored using chi-square and t-tests (**Table 11).**

Table 11: Differences between White-British and minoritized ethnic groups across outcome measures (unadjusted, female sub-sample)

| **Outcome measure** | **White-British** | **All minorized ethnicities** | ***X^2^*** |
| --- | --- | --- | --- |
| Reliably recovered | 47% | 41% | *X*^2^(1, N=66293) = 218.95, *p*<.001 |
| Deteriorated | 7% | 8% | *X*^2^(1, N=66293) = 49.63, *p*<.001 |
| Dropped out | 31% | 31% | -- |
| **Change scores** | **White-British** | **All minoritized ethnicities** | **t-test** |
| Change scores on the PHQ-9 (from baseline to end of treatment) | *N*=29161, *M*=5.80, *SD*=6.27 | *N*=37129, *M*=5.73, *SD* = 6.58 | *t*(63872) = -1.27, *p*=.204 |
| Change scores on the GAD-7 (from baseline to end of treatment) | *N*=29161, *M*=5.65, *SD*=5.81 | *N*=37118, *M*=5.21, *SD*=5.98 | *t*(63381.3) = -9.57, *p*<.001 |

## **Differences in treatment outcomes between females from minoritized ethnic groups and White-British females independent of particular patient characteristics and factors**

When controlling for socio-demographic and clinical variables, White-British females were significantly more likely to recover (OR=1.07 (95% CI: 1.03-1.11), *p*=.001) and more likely to improve (OR-1.08 (95% CI: 1.04-1.13), *p*=<.001) than females in the amalgamated minoritized ethnicity group. Females in the minoritized ethnicity group were significantly more likely to deteriorate after treatment, than White-British females (OR=0.84 (95% CI: 0.78-0.91), *p*=001 but no significant differences were observed regarding drop-out (OR-1.01 (95% CI: 0.97-1.06), *p*=.540) (**Table 12**).

Table 12: RQ2 using 2 ethnicity categories (White-British females and minoritized ethnicity females)

| **Model** | **Variables** | **Reliable recovery: odds ratio (95% CI), *p*-value** | **Reliable improvement: odds ratio (95% CI), *p*-value** | **Reliable deterioration: odds ratio (95% CI), *p*-value** | **Drop-out: odds ratio (95% CI), *p-*value** |
| --- | --- | --- | --- | --- | --- |
| Model 1 | Ethnicity | 1.26 (1.22-1.30), *p*<.001 | 1.15 (1.12-1.20), *p*<.001 | 0.81 (0.77-0.86), *p*<.001 | 0.94 (0.91-0.98), *p*=.001 |
| Model 2 | (Model 1) + Service | 1.27 (1.24-1.32), *p*<.001 | 1.16 (1.12-1.20), *p*<.001 | 0.80 (1.75-0.84), *p*<.001 | 0.90 (0.87-0.93), *p*<01 |
| Model 3 | (Model 2) + Age, LTC status | 1.25 (1.22-1.30), *p*<.001 | 1.17 (1.13-1.21) *p*<.001 | 0.82 (0.78-0.87), *p*<.001 | 0.93 (0.90-0.97), *p*<.001 |
| Model 4 | (Model 3) + Baseline severity PHQ9, Baseline severity GAD7, Baseline severity WSAS, diagnosis/presenting problem, social phobia, agoraphobia, specific phobia | 1.12 (1.08-1.16), *p*<.001 | 1.13 (1.09-1.17) *p*<.001 | 0.80 (0.75-0.86), *p*<.001 | 1.00 (0.96-1.04), *p*=.885 |
| Model 5 | (Model 4) + IMD, employment status, Medication status | 1.07 (1.03-1.11), *p*=.001 | 1.08 (1.04-1.13) *p*<.001 | 0.84 (0.78-0.91), *p*<.001 | 1.01 (0.97-1.06), *p*=.540 |

## **Differences in treatment outcomes between minoritized ethnic females (using Office for National Statistics ethnicity categories) and White-British females, independent of particular patient characteristics and factors**

This question explored differences in outcomes between White-British females and females from different minoritized ethnic groups using the ONS ethnicity categories, allowing for comparisons that are specific to more nuanced categories of ethnicity (**Table 1**3).

Table 13: RQ2.1 using ONS categories of ethnicity; White-British = reference category (female sub-group)

| **Model** | **Variables** | **ONS ethnicity categories** | **Reliable recovery: odds ratio (95% CI) *p*-value** | **Reliable improvement: odds ratio (95% CI) *p*-value** | **Reliable deterioration: odds ratio (95% CI) *p*-value** | **Drop-out: odds ratio (95% CI) *p-*value** |
| --- | --- | --- | --- | --- | --- | --- |
| Model 1 | Ethnicity | Asian  Black  Chinese  Mixed  Other  White-other | 0.73 (0.69-0.76) *p*<.001  0.83 (0.79-0.87) *p*<.001  1.07 (0.01-1.27) *p*=.412  0.80 (0.75-0.86) *p*<.001  0.60 *0.55-0.65) *p*<.001  0.83 (0.80-0.87) *p*<.001 | 0.83 (0.79-0.88) *p*<.001  0.92 (0.88-0.98) *p*=.005  1.06 (0.88-1.28) *p*=.539  0.89 (0.83-0.95) *p*=.001  0.68 (0.62-0.74) *p*<.001  0.88 (0.84-0.91) *p*<.001 | 1.29 (1.17-1.41) *p*<.001  1.26 (1.15-1.38) *p*<.001  1.14 (0.83-1.56) *p*=.428  1.25 (1.11-1.41) *p*<.001  1.43 (1.40-1.64) *p*<.001  1.16 (1.08-1.25) *p*<.001 | 1.22 (1.16-1.30) *p*<.001  1.25 (1.18-1.32) *p*<.001  0.77 (0.63-0.94) *p*=.012  1.24 (1.16-1.34) *p*<.001  1.16 (1.06-1.28) *p*=.001  0.86 (0.82-0.90) *p*<.001 |
| Model 2 | (Model 1) + Service | Asian  Black  Chinese  Mixed  Other  White-other | 0.70 (0.67-0.74) *p*<.001  0.81 (0.77-0.86) *p*<.001  1.09 (0.92-1.30) *p*=.301  0.80 (0.75-0.85) *p*<.001  0.59 (0.54-0.65) *p*<.001  0.83 (0.80-0.87) *p*<.001 | 0.82 (0.77-0.87) *p*<.001  0.91 (0.86-0.96) *p*=.001  1.06 (0.88-1.28) *p*=.541  0.88 (0.82-0.94) *p*<.001  0.66 (0.61-0.72) *p*<.001  0.86 (0.83-0.90) *p*<.001 | 1.32 (1.20-1.46) *p*<.001  1.26 (1.15-1.38) *p*<.001  1.18 (0.86-1.62) *p*=.308  1.27 (1.13-1.43) *p*<.001  1.47 (1.27-1.69) *p*<.001  1.19 (1.11-1.28) *p*<.001 | 1.18 (1.11-1.25) *p*<.001  1.25 (1.18-1.32) *p*<.001  0.86 (0.70-1.06) *p*=.167  1.30 (1.21-1.40) *p*<.001  1.30 (1.19-1.43) *p*<.001  0.94 (0.90-0.99) *p*=.009 |
| Model 3 | (Model 2) + Age, LTC status | Asian  Black  Chinese  Mixed  Other  White-other | 0.71 (0.67-0.75) *p*<.001  0.82 (0.78-0.87) *p*<.001  1.13 (0.95-1.35) *p*=.178  0.83 (0.53-0.64) *p*<.001  0.58 (0.53-0.64) *p*<.001  0.85 (0.81-0.88) *p*<.001 | 0.81 (0.76-0.86) *p*<.001  0.92 (0.87-0.97) *p*=.003  1.07 (0.88-1.30) *p*=.515  0.90 (0.83-0.97) *p*=.004  0.65 (0.59-0.71) *p*<.001  0.87 (0.83-0.91) *p*<.001 | 1.34 (1.21-1.47) *p*<.001  1.25 (1.14-1.38) *p*<.001  1.19 (0.85-1.38) *p*=.304  1.26 (1.11-1.42) *p*<.001  1.47 (1.26-1.70) *p*<.001  1.16 (1.07-1.26) *p*<.001 | 1.13 (1.06-1.20) *p*<.001  1.23 (1.16-1.30) *p*<.001  0.81 (0.66-1.00) *p*=.053  1.18 (1.10-1.28) *p*<.001  1.32 (1.19-1.45) *p*<.001  0.91 (0.87-0.96) *p*<.001 |
| Model 4 | (Model 3) + Baseline severity PHQ9, Baseline severity GAD7, Baseline severity WSAS, diagnosis/presenting problem, social phobia, agoraphobia, specific phobia | Asian  Black  Chinese  Mixed  Other  White-other | 0.82 (0.77-0.87) *p*<.001  0.99 (0.94-1.05) *p*=.838  1.23 (1.01-1.50) *p*=.042  0.91 (0.84-0.98) *p*=.015  0.70 (0.64-0.77) *p*<.001  0.89 (0.85-0.94) *p*<.001 | 0.85 (0.80-0.91) *p*<.001  1.01 (0.95-1.08) *p*=.700  1.26 (1.00-1.57) *p*=.051  0.93 (0.85-1.00) *p*=.067  0.68 (0.62-0.75) *p*<.001  0.86 (0.87-1.09) *p*<.001 | 1.42 (1.28-1.58) *p*<.001  1.17 (1.05-1.30) *p*=.004  0.95 (0.65-1.39) *p*=.791  1.17 (1.01-1.34) *p*=.032  1.53 (1.30-1.81) *p*<.001  1.13 (1.04-1.24) *p*=.005 | 1.04 (0.98-1.12) *p*=.207  1.12 (1.05-1.19) *p*=.001  0.79 (0.63-1.00) *p*=.047  1.11 (1.07-1.32) *p*=.016  1.19 (1.07-1.32) *p*=.002  0.87 (0.83-0.92) *p*<.001 |
| Model 5 | (Model 4) + IMD, employment status, Medication status | Asian  Black  Chinese  Mixed  Other  White-other | 0.86 (0.81-0.92) *p*<.001  1.06 (0.99-1.12) *p*=.094  1.25 (1.02-1.53) *p*=.035  0.95 (0.88-1.03) *p*=.241  0.78 (0.70-0.86) *p*<.001  0.92 (0.88-0.97) *p*=.001 | 0.89 (0.82-0.94) *p*<.001  1.08 (1.01-1.16) *p*=.024  1.27 (1.00-1.62) *p*=.046  0.96 (0.88-1.05) *p*=.355  0.74 (0.67-0.83) *p*<.001  0.88 (0.84-0.93) *p*<.001 | 1.36 (1.22-1.53) *p*<.001  1.08 (0.96-1.21) *p*=.200  0.99 (0.66-1.49) *p*=.980  1.18 (1.02-1.37) *p*=.024  1.43 (1.20-1.71) *p*<.001  1.14 (1.03-1.25) *p*=.007 | 1.02 (0.95-1.10) *p*=.502  1.08 (1.01-1.16) *p*=.027  0.81 (0.64-1.03) *p*=.087  1.07 (0.98-1.17) *p*=.134  1.13 (1.01-1.27) *p*=.032  0.86 (0.82-0.91) *p*<.001 |

Least likely to recover when compared to White-British females, were those in the ‘Other’ group (OR=0.78 [95% CI: 0.70-0.86], *p*<.001), followed those of Asian ethnicity (OR= 0.86 [95% CI:0.81-0.92], *p*<.001). Chinese females showed significantly increased likelihood of recovery compared to White-British females, even when controlling for socio-demographic and clinical factors (OR=1.25 [95% CI:1.02-1.53), *p*=.035).

Females in the Asian, ‘Other’ and Other-White groups were significantly less likely to improve following treatment (OR=0.89 [95% CI:0.82-0.94], *p*<.001; OR=0.74 [95% CI:0.67-0.83, *p*<.001; OR=0.88 [95% CI:0.84-0.93], *p<.*001). Chinese and Black females were the only groups significantly more likely to improve than White-British females (OR=1.27 [95% CI: 1.00-1.62], *p*=.046; OR=1.08 [95% CI:1.01-1.16], *p=*.024).

When controlling for socio-demographic and clinical variables, all minoritized ethnic groups showed significantly more likelihood of deterioration following treatment compared to White-British females, with the exception of females belonging to Black (OR=1.08 (95% CI: 0.96-1.21) *p*=.200) and Chinese (OR=0.99 (94% CI: 0.66-1.49) *p*=.980) ethnic groups. Females belonging to the Asian and ‘Other’ ethnic groups showed the most likelihood of worsening symptoms following treatment compared to White-British females (Asian: OR=1.36 [95% CI: 1.22-1.53], *p*<.001; ‘Other’: OR= 1.43 [95%: 1.20-1.71], *p*<.001).

Females belonging to Black and ‘Other’ groups showed significantly higher odds of drop-out than White British women when controlling for socio-demographic and clinical variables (Black: OR=1.08 [95% CI:1.01-1.16], *p*=.027; ‘Other’: OR=1.13 [95% CI: 1.01-1.27], *p*=.032). Those in the White-other group were the only group to show significantly less likelihood of dropping out of treatment (OR=0.86 [95% CI:0.82-0.91], *p*<.001).

### Employment and IMD

An additional analysis (Model 6, **Table 14**) was performed to explore the impact of removing IMD and employment as potential confounding variables, on reliable recovery outcomes.

Table 14: Linear regression model removing IMD and employment status as potential confounders (female subsample)

| **Model** | **Variables** | **ONS ethnicity categories** | **Reliable recovery: odds ratio (95% CI) *p*-value** |
| --- | --- | --- | --- |
| Model 5 | (Model 4) + IMD, employment status, Medication status | Asian  Black  Chinese  Mixed  Other  White-other | 0.86 (0.81-0.92) *p*<.001  1.06 (0.99-1.12) *p*=.094  1.25 (1.02-1.53) *p*=.035  0.95 (0.88-1.03) *p*=.241  0.78 (0.70-0.86) *p*<.001  0.92 (0.88-0.97) *p*=.001 |
| Model 6 | (Model 4) + Medication status | Asian  Black  Chinese  Mixed  Other  White-other | 0.81 (0.76-0.62) *p*<.001  0.98 (0.92-1.04) *p*=.500  1.22 (0.99-1.49) *p*=.062  0.90 (0.83-0.98) *p*=.012  0.70 (0.63-0.78) *p*<.001  0.88 (0.84-0.92) *p*<.001 |

Removal of IMD and employment status as variables in the model resulted in significantly lower odds of reliable recovery for Mixed females relative to White-British females. Removing these two variables form the model erased the significantly higher odds of reliable recovery observed for Chinese females in Model 5 (**Table 14**).

## **Organization-level factors**

Logistic regression models with interactions terms were used to explore the potential differential effects of organization-level factors on outcomes for different ethnic groups, whilst controlling for socio-demographic and clinical variables.

Table 15: Results from the likelihood-ratio test used to compare the adjusted model (model 5) with the inclusion of an interaction term

| **Organization-level variable** | **Reliable recovery** | **Reliable improvement** | **Deterioration** | **Dropout** |
| --- | --- | --- | --- | --- |
| Number of sessions | *X^2^* (6) = 20.35, *p*=.002 | *X^2^* (6) = 8.16, *p*=.227 | *X^2^* (6) = 9.13, *p*=.016 | *X^2^* (6) = 9.85, *p*=.013 |
| Referral source (GP/self-referral/other) | *X^2^* (12) = 26.01, *p*=.011 | *Omitted | *Omitted | *X^2^* (12) = 17.39, *p*=.136 |
| Number of face-to-face sessions | *X^2^* (6) = 17.01 *p*=.009 | *X^2^* (6) = 8.81, *p*=.185 | *X^2^* (6) = 10.72, *p*=.098 | *X^2^* (6) = 7.23 *p*=.300 |
| Number of telephone sessions | *X^2^* (6) = 5.48, *p*=.484 | *X^2^* (6) = 2.68, *p*=.857 | *X^2^* (6) = 9.38, *p*=.153 | *X^2^* (6) = 3.39 *p*=.759 |
| Number of video call sessions | *X^2^* (15) = 13.01, *p*=.023 | *Omitted | *Omitted | *Omitted |
| Number of days from referral to assessment (primary wait) | *X^2^* (14) = 20.48, *p*=.116 | *X^2^* (6) = 4.53, *p*=.605 | *X^2^* (6) = 3.37, *p*=.761 | *X^2^* (6) = 10.20, *p*=.117 |
| Number of days assessment to treatment (secondary wait) | *X^2^* (6) = 1.61, *p*=.952 | *X^2^* (6) = 3.39, *p*=.759 | *X^2^* (6) = 5.73, *p*=.454 | *X^2^* (6) = 5.82 *p*=.444 |
| Number of high intensity treatment sessions | *X^2^* (6) = 12.23, *p*=.057 | *X^2^* (6) = 7.01, *p*=.319 | *X^2^* (6) = 12.05 *p*=.061 | *X^2^* (6) = 1.93 *p*=.926 |
| **Missing values led to unequal observations between models, restricting comparison using the likelihood-ratio test.* | | | | |

Inclusion of interaction terms resulted in improvement in several of the models (**Table 15**). The results from the improved models (i.e., *p*<.005) are provided below.

### **Reliable recovery**

Table 16: Results from logistic regression models with interactions for the female subsample – reliable recovery outcome. Included are the factors for each ethnic group that resulted in odds ratios with a p-value of <.05. (reference category = White-British females)

| **Reliable recovery** | | | |
| --- | --- | --- | --- |
| **Organization-level variables** | **Ethnic group** | | |
|  | **Mixed** | **Other** | **White-other** |
| Number of sessions | OR=1.02 (CI:1.01-1,04), *p*=.011 | -- | OR=0.99 (CI:0.98-1.00), *p*=.026 |
| Referral to assessment wait time (weeks) | -- | -- | OR=1.00 (CI: 0.99-1.00), p=.028 |
| Form used to provide treatment (number of face-to-face sessions) | OR=1.02 (CI:1.00-1.04), *p*=.013 | -- | -- |
| Form used to provide treatment (number of telephone sessions) | -- | -- | OR=0.97 (CI:0.95-1.00), *p*=.037 |
| Number of high intensity treatment sessions | OR=1.02 (CI:1.00-1.04), *p*=.007 | -- | -- |

There was a small but significant interaction between specific ethnicity groups and the number of treatment sessions received. Specifically, a higher number of sessions was associated with a small increase in the odds of reliable recovery for Mixed (OR=1.02 [95 % CI: 1.01-1.04], *p*=.011) females relative to White-British females. Conversely, a higher number of sessions for White-other females was associated with a very slight decrease in odds of recovery (OR=0.99 [95% CI: 0.98-1.00], *p*=.026) females, compared to White-British females.


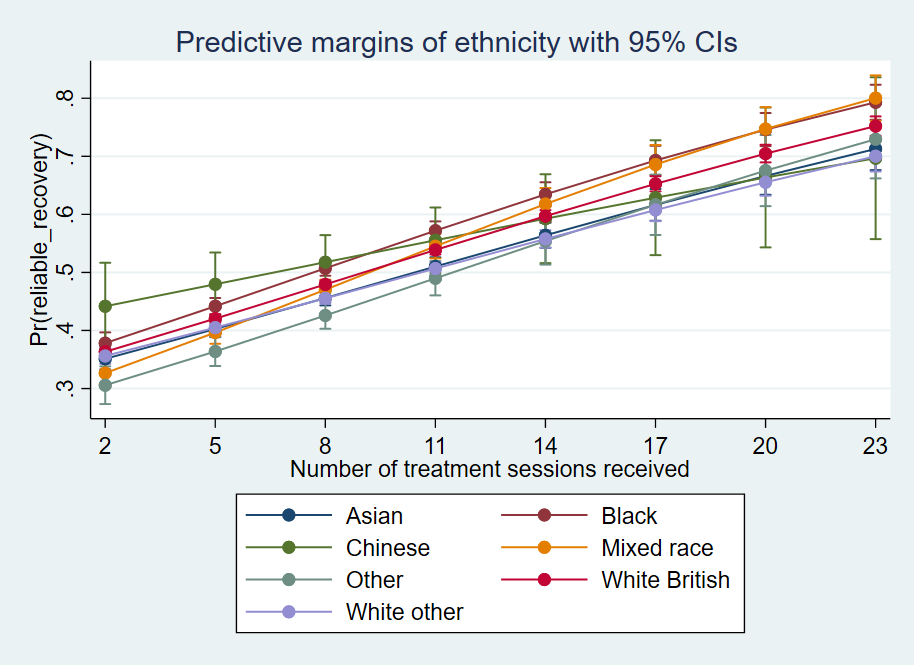


Figure 12: Graph to show ethnic group and number of treatment sessions received as predictors of reliable recovery (female sub-sample)

The number of high intensity treatment sessions was associated with a small significant increase in odds of reliable recovery for females in the Mixed group, relative to White British females (OR=1.02 [95% CI: 1.00-1.04], *p*=.007).


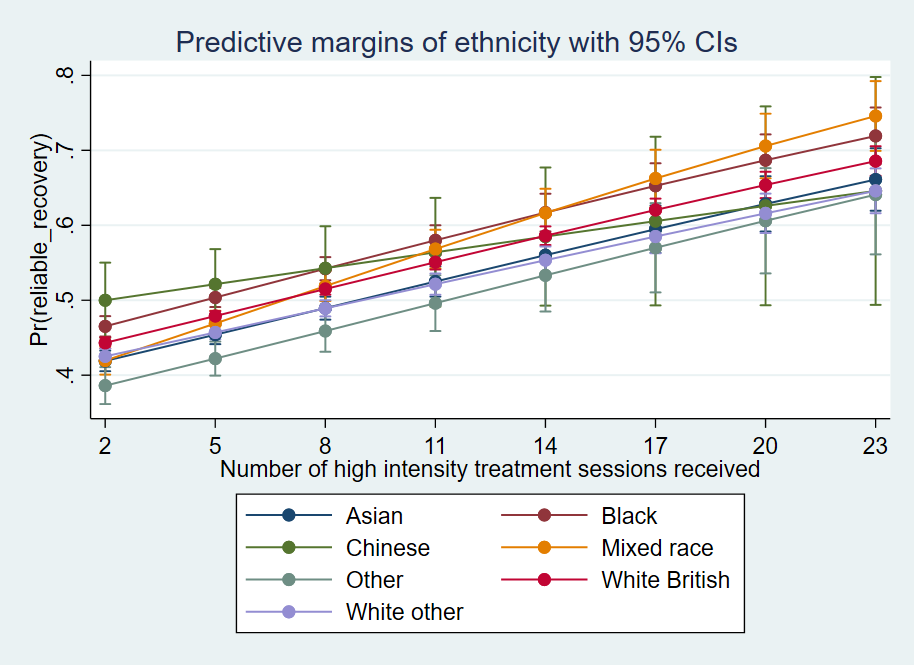


Figure 13: Graph to show ethnic group and number of high intensity treatment sessions received as predictors of reliable recovery (female sub-sample)

There were significant interactions between specific ethnicity groups and the number of face-to-face sessions received. Specifically, a higher number of face-to-face sessions was associated with small but significantly increased odds of reliable recovery for Mixed females, relative to White-British females OR=1.02 [95% CI:1.00-1.04], *p*=.013).


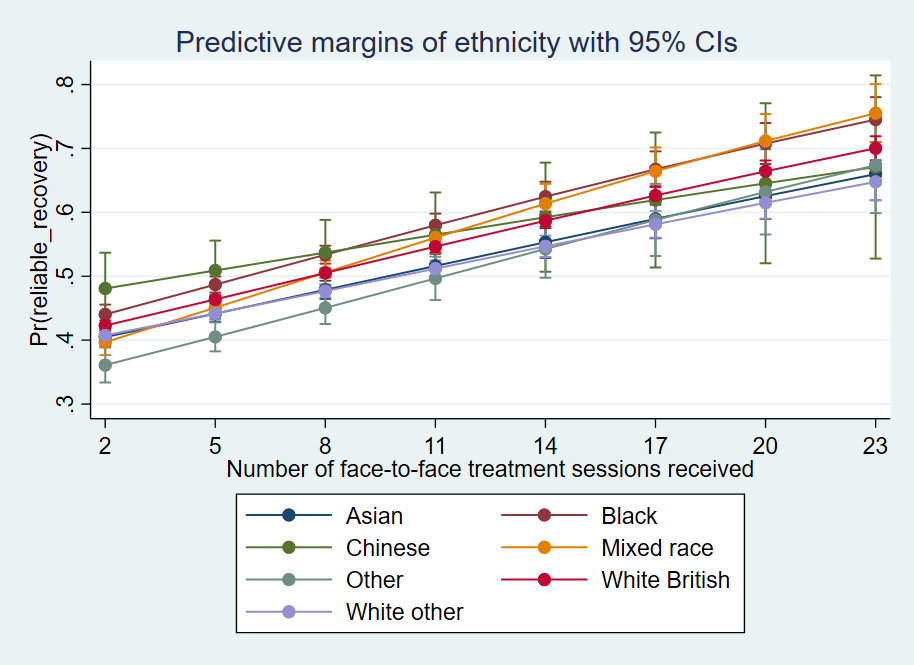


Figure 14: Graph to show ethnic group and number of face-to-face treatment sessions received as predictors of reliable recovery (female sub-sample)

There was a significant interaction between belonging to the White-other group and the number of telephone sessions received; the number of telephone sessions was associated with decreased odds of reliable recovery in this group, relative to White-British females (OR=0.97 [95% CI:0.95-1.00], *p*=.037).


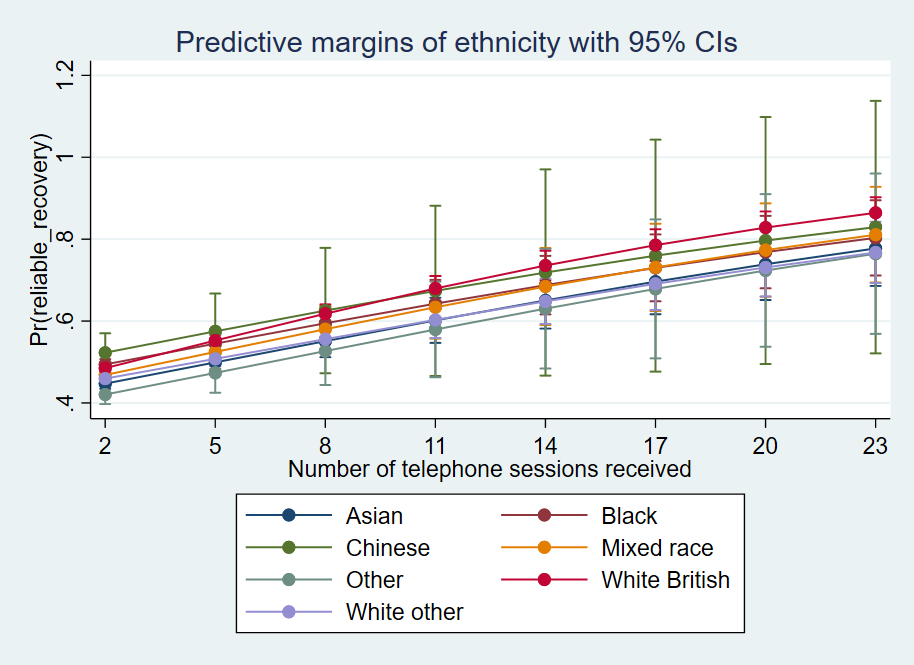


Figure 15: Graph to show ethnic group and number of telephone treatment sessions received as predictors of reliable recovery (female sub-sample)

### **Reliable improvement**

Table 17:Results from logistic regression models with interactions for the female subsample – reliable improvement outcome. Included are the factors for each ethnic group that resulted in odds ratios with a p-value of <.05. (reference category = White-British females)

| **Reliable improvement** | | | | |
| --- | --- | --- | --- | --- |
| **Organisation-level variables** | **Ethnic group** | | | |
|  | **Asian** | **Chinese** | **Other** | **White-other** |
| Method of access (GP referral) | -- | -- | OR=0.55 (CI:0.32--.96), *p*=.037* | -- |
| Method of access (self-referral) | -- | -- | OR=0.56 (CI:0.32-1.11), *p*=0.037* | -- |
| Form used to provide treatment (number of face-to-face sessions) | -- | -- | -- | OR=0.99 (CI:0.98-1.00), *p*=.033* |
| Number of high intensity treatment sessions | -- | OR=0.95 (CI:0.91-1.00), *p*=.037* | -- | -- |

The number of high intensity treatment sessions was associated with significantly decreased odds of reliable improvement for Chinese females relative to White-British females (OR=0.95 [95% CI:0.91-1.00], *p*=.037).


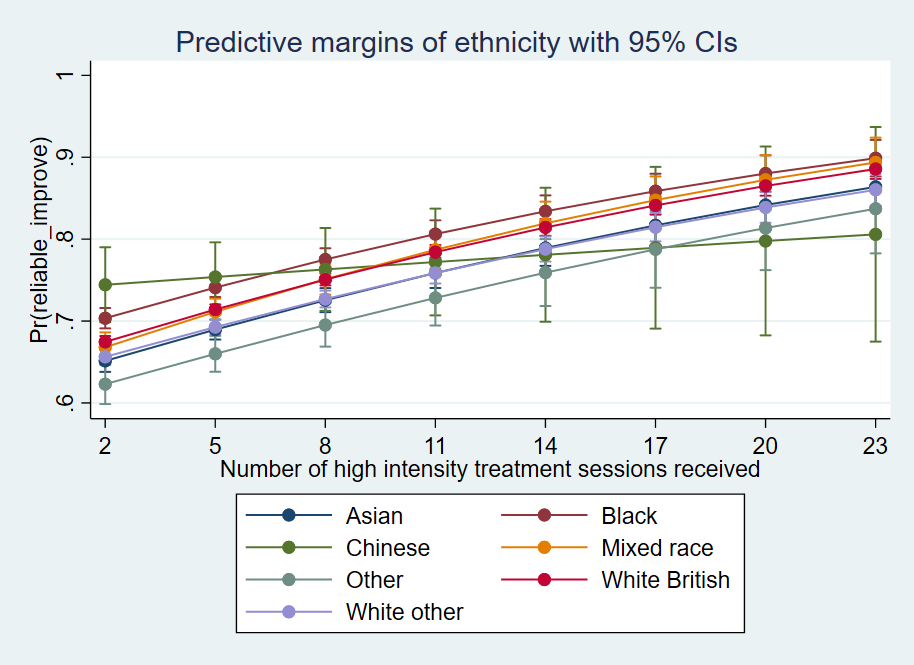


Figure 16: Graph to show ethnic group and number of high intensity treatment sessions as predictors of reliable improvement (female subsample)

### **Deterioration**

Table 18: Results from logistic regression models with interactions for the female subsample – deterioration outcome. Included are the factors for each ethnic group that resulted in odds ratios with a p-value of <.05. (reference category = White-British

| **Deterioration** | |
| --- | --- |
| **Organization-level variables** | **Ethnic group** |
|  | **Chinese** |
| Number of sessions | OR=1.10 (CI:1.01-1.20), *p*=.022 |
| Form used to provide treatment (number of face-to-face sessions) | OR=1.09 (CI:1.02-1.17), *p*=.013 |
| Number of high intensity treatment sessions | OR=1.10 (CI:1.03-1.17), *p*=.006 |

Interestingly, the odds of deterioration following a course of treatment were associated with the number of sessions for Chinese females relative to White-British females (OR=1.10 [95% CI:1.02-1.20], *p*=.020).


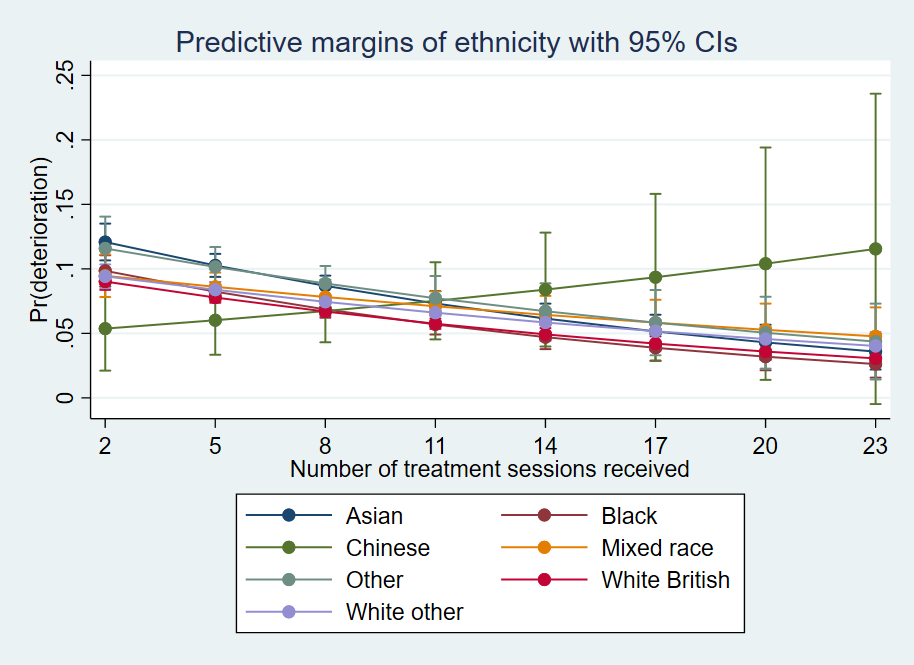


Figure 17: Graph to show ethnic group and number of treatment sessions received as predictors of deterioration (female subsample)

Chinese females also showed significantly increased odds of deterioration with higher numbers of high intensity treatment sessions received, relative to White-British females. (OR=1.10 [95% CI:1.03-1.17], *p*=.006).


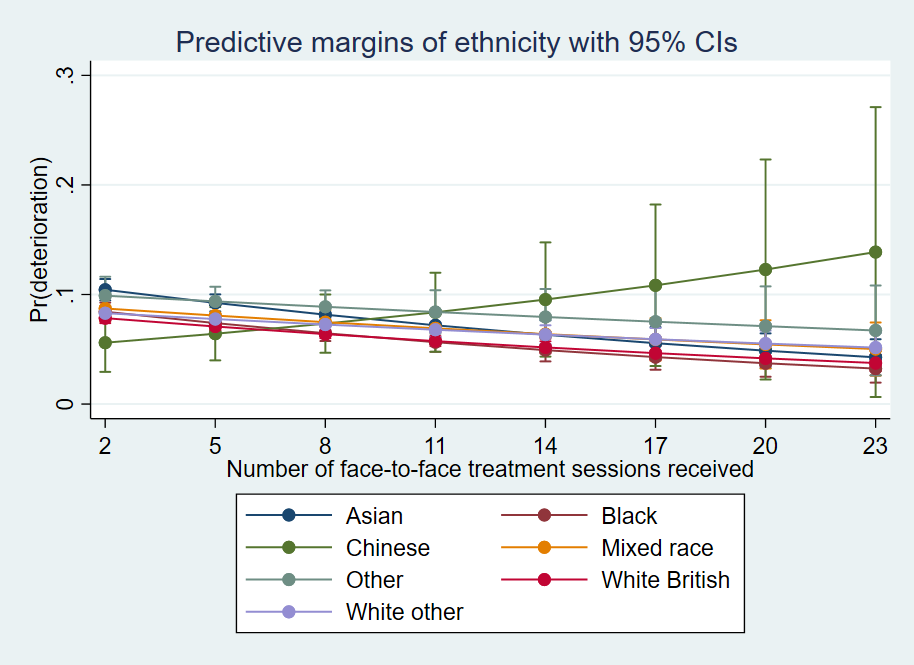


Figure 18: Graph to show ethnic group and number of face-to-face treatment sessions received as predictors of deterioration (female subsample)


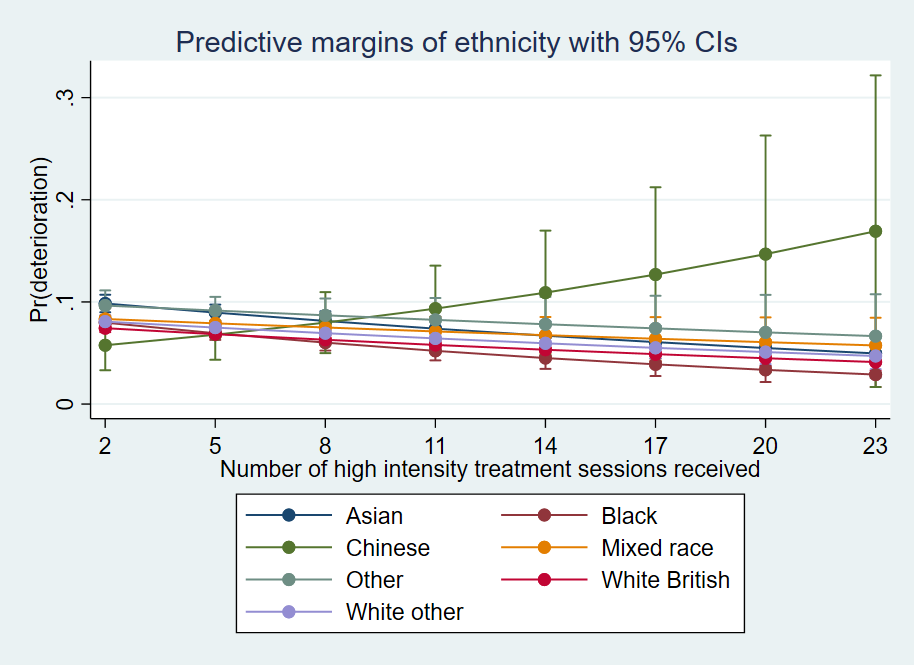


Figure 19: Graph to show ethnic group and number of high intensity treatment sessions received as predictors of deterioration (female subsample)

### **Drop-out**

Table 19: Results from logistic regression models with interactions for the female subsample – drop-out outcome. Included are the factors for each ethnic group that resulted in odds ratios with a p-value of <.05. (reference category = White-British females)

| **Drop-out** | | |
| --- | --- | --- |
| **Organization-level variables** | **Ethnic group** | |
|  | **Black** | **White-other** |
| Number of sessions | OR=0.97 (CI:0.94-1.00), *p*=.025* | OR=0.97 (CI: 0.95-0.99), *p*=.013* |
| Form used to provide treatment (number of face-to-face sessions) | OR=0.98 (CI:0.96-1.00), *p*=.046* | -- |

A higher number of treatment sessions was associated with significantly decreased odds of drop-out for Black females, (OR=0.97 [95% CI: 0.94-1.00], *p*=.025) and White-other females (OR=0.97 [95% CI: 0.95-0.99], *p*=.013) relative to White-British females.


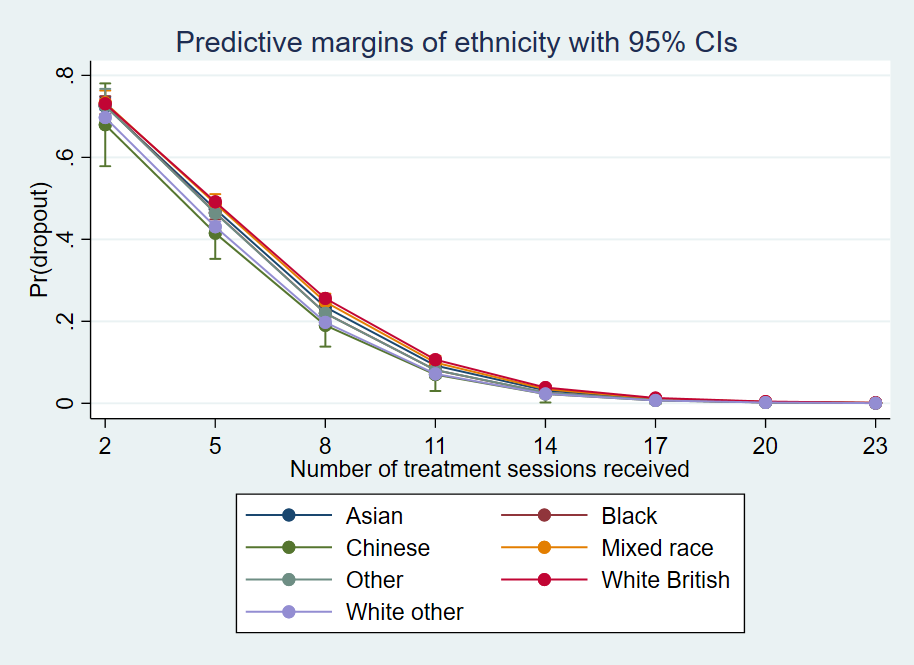


Figure 20: Graph to show ethnic group and number of treatment sessions received as predictors of drop- out (female subsample)

There was a small but significant interaction between ethnicity and the number of face-to-face treatment sessions received. A higher number of face-to-face sessions was associated with decreased odds of drop-out for Black females, relative to White-British female (OR=0.98 [95% CI:0.96-1.00), *p*=.046).


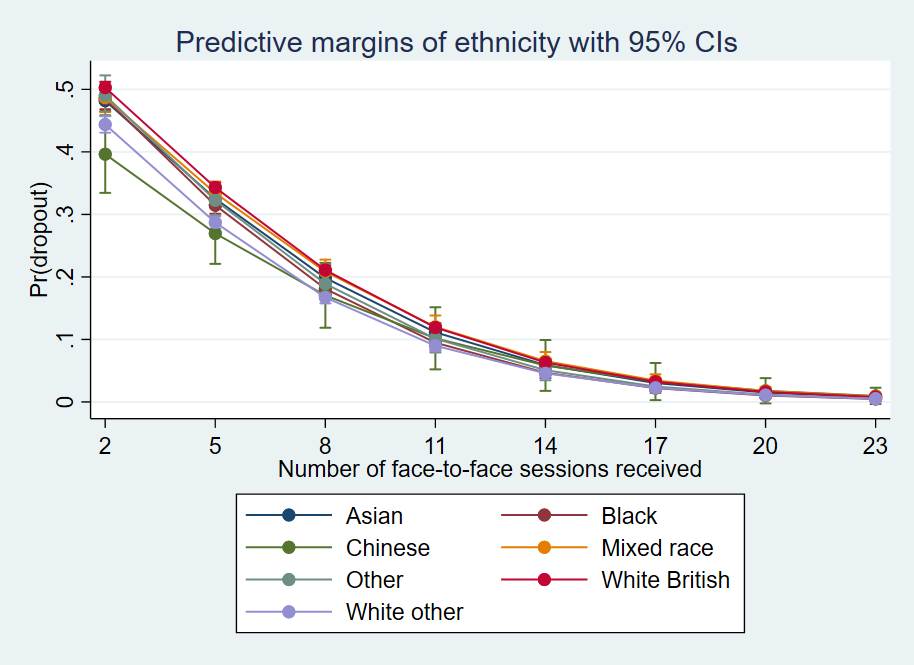


Figure 21: Graph to show ethnic group and number of face-to-face treatment sessions received as predictors of drop- out (female subsample)

# Appendix 6: Results - male sub-group analyses

## **Comparison of baseline demographic and clinical characteristics between ethnic groups**

The largest ethnic category was White-British (50%) followed by males of White-other ethnicity (19%). Asian males accounted for 13% of the sample while the smallest group was of Chinese males who made up less than 1% of the sub-sample (**Table 20**).

Table 20: Ethnicity breakdown - male subsample

| **Ethnicity ONS** | **N** | **%** |
| --- | --- | --- |
| White British | 15831 | 50.23 |
| Asian | 4043 | 12.83 |
| Black | 2801 | 8.89 |
| Chinese | 166 | 0.53 |
| Mixed | 1581 | 5.02 |
| Other | 1178 | 3.74 |
| White - other | 5915 | 18.77 |
| Total | 31515 | |

As with the whole sample and female sub-samples, demographic and clinical characteristics between two ethnic groups (White-British and all minoritized ethnic groups) were compared (**Table 20**). Comparison of continuous variables was performed using t-tests, chi-square tests were used for categorical variables.

Table 21: Comparison of baseline demographic and clinical information (continuous and categorical variables) between ethnic groups (male sub-sample)

| **Continuous variables** | | **White-British** | | | | **All minoritized ethnic groups** | | | | | | **Difference** | |
| --- | --- | --- | --- | --- | --- | --- | --- | --- | --- | --- | --- | --- | --- |
|  |  | N | Mean | | **Sd** | **N** | | **Mean** | | **Sd** | | ***p*-value** | |
| PHQ-9 | | 15831 | 14.7 | | 5.58 | 15682 | | 16.11 | | 5.66 | | <.001 | |
| GAD-7 | | 15830 | 13.38 | | 4.45 | 15675 | | 14.30 | | 4.49 | | <.001 | |
| WSAS 1 | | 12975 | 5.36 | | 2.71 | 13386 | | 5.74 | | 2.83 | | <.001 | |
| WSAS 2 | | 12975 | 3.43 | | 2.37 | 13387 | | 3.88 | | 2.51 | | <.001 | |
| WSAS 3 | | 12975 | 4.27 | | 2.40 | 13378 | | 4.66 | | 2.51 | | <.001 | |
| WSAS 4 | | 12974 | 3.50 | | 2.47 | 13378 | | 4.01 | | 2.59 | | <.001 | |
| WSAS 5 | | 12974 | 4.05 | | 2.40 | 13377 | | 3.37 | | 2.49 | | <.001 | |
| Social phobia | | 15586 | 3.14 | | 2.50 | 15263 | | 3.66 | | 2.68 | | <.001 | |
| Specific phobia | | 15581 | 1.88 | | 2.52 | 15256 | | 2.47 | | 2.80 | | <.001 | |
| Agoraphobia | | 15578 | 2.43 | | 2.62 | 15256 | | 2.94 | | 2.81 | | <.001 | |
| Number of LI sessions | | 15831 | 2.93 | | 2.78 | 15684 | | 2.88 | | 2.73 | | .080 | |
| Number of HI sessions | | 15831 | 4.68 | | 5.57 | 15684 | | 4.26 | | 5.19 | | <.001 | |
| Days waited from referral to assessment | | 15828 | 3.67 | | 4.56 | 15677 | | 3.84 | | 4.70 | | .001 | |
| Days waited from assessment to treatment | | 14801 | 8.20 | | 7.98 | 14467 | | 8.92 | | 5.56 | | <.001 | |
| Age | | 15831 | 39.41 | | 14.10 | 15684 | | 37.93 | | 12.66 | | <.001 | |
| **Categorical variables** | | | | **White-British** | | | | | **All minoritized ethnic groups** | | | | ***p*-value** |
|  |  |  |  | **N** | | | **%** | | **N** | | **%** | |  |
| Employment | Employed | | | 11777 | | | 74.97 | | 10395 | | 67.36 | | <.001 |
|  | Unemployed | | | 3931 | | | 25.03 | | 5104 | | 32.93 | |  |
| Existing long-term condition | Yes | | | 3988 | | | 26.65 | | 4150 | | 28.37 | | .001 |
|  | No | | | 10977 | | | 73.35 | | 10479 | | 71.63 | |  |
| IMD decile | Low | | | 8465 | | | 85.24 | | 9249 | | 90.22 | | <.001 |
|  | High | | | 1466 | | | 14.76 | | 1003 | | 9.8 | |  |
| Diagnosis/presenting problem | Depression | | | 5811 | | | 37.52 | | 6424 | | 41.86 | | <.001 |
|  | Anxiety disorder | | | 1983 | | | 12.81 | | 1983 | | 12.92 | |  |
|  | MAAD | | | 849 | | | 5.49 | | 903 | | 5.88 | |  |
|  | PTSD | | | 245 | | | 1.58 | | 759 | | 4.95 | |  |
| *GAD-7 = generalised anxiety disorder scale (anxiety symptom measure)*  *HI = high intensity treatment*  *IMD = indices of multiple deprivation*  *LI = low intensity treatment*  *MADD = mixed anxiety and depression*  *PHQ-9 = patient health questionnaire (depression symptom measure)*  *PTSD = post-traumatic stress disorder*  *WSAS = work and social adjustment scale (items 1-5)* | | | | | | | | | | | | | |

## **Differences in unadjusted outcomes between males from minoritized ethnicity groups and White British males**

Differences in a variety of treatment outcomes between an amalgamated sub-group of males from minoritized ethnic backgrounds compared to White-British males were explored using chi-square and t-tests (**Table 22**).

Table 22: Differences between White-British and minoritized ethnic groups across outcome measures (unadjusted, male sub-sample)

| **Outcome measure** | **White-British** | **All minorized ethnicities** | ***X^2^*** |
| --- | --- | --- | --- |
| Reliably recovered | 49% | 42% | *X^2^*(1, N=31515) = 174.27, *p*<.001 |
| Deteriorated | 7% | 10% | *X*^2^(1, N=31515) = 44.25, *p*<.001 |
| Dropped out | 28% | 33% | *X*^2^(1, N=28200) = 79.83, *p*<.001 |
| **Change scores** | **White-British** | **All minoritized ethnicities** | **t-test** |
| Change scores on the PHQ-9 (from baseline to end of treatment) | N=15831 M=5.84, SD=6.25 | N=15682, M=5.67, SD 6.73 | *t*(31296.8) = -2.277, *p*=.023 |
| Change scores on the GAD-7 (from baseline to end of treatment) | N=15830, M=5.49, SD=5.68 | N=15675, M=5.11, SD=5.94 | *t*(31409.7) = -5.852, *p*<.001 |

## **Differences in treatment outcomes between males from minoritized ethnic groups and White-British males independent of particular patient characteristics and factors**

White British males were consistently significantly more likely to recover and more likely to improve than males in the minoritized ethnicity group. Males in the minoritized ethnicity group were significantly more likely to deteriorate after treatment and to drop out, than White British males (**Table 23).** This was the case even controlling for socio-demographic and clinical variables.

Table 23: RQ1 using 2 ethnicity categories (White-British males and minoritized ethnicity males)

| **Model** | **Variables** | **Reliable recovery: odds ratio (95% CI) *p*-value** | **Reliable improvement: odds ratio (95% CI) *p*-value** | **Reliable deterioration: odds ratio (95% CI) *p*-value** | **Drop-out: odds ratio (95% CI) *p*-value** |
| --- | --- | --- | --- | --- | --- |
| Model 1 | Ethnicity | 1.35 (1.29-1.41) *p*<.001 | 1.20 (1.14-1.26) *p*<.001 | 0.75 (0.69-0.82) *p*<.001 | 0.79 (0.75-0.83) *p*<.001 |
| Model 2 | (Model 1) + Service | 1.36 (130-1.42) *p*<.001 | 1.20 (1.14-1.26) *p*<.001 | 0.75 (0.69-0.82) *p*<.001 | 0.78 (0.74-0.82) *p*<.001 |
| Model 3 | (Model 2) + Age, LTC status | 1.36 (1.30-1.42) *p*<.001 | 1.21 (1.16-1.28) *p*<.001 | 0.75 (0.69-0.82) *p*<.001 | 0.79 (0.75-0.84) *p*<.001 |
| Model 4 | (Model 3) + Baseline severity PHQ9, Baseline severity GAD7, Baseline severity WSAS, diagnosis/presenting problem, social phobia, agoraphobia, specific phobia | 1.16 (1.10-1.22) *p*<.001 | 1.15 (1.09-1.22) *p*<.001 | 0.72 (0.65-0.80) *p*<.001 | 0.85 (0.80-0.90) |
| Model 5 | (Model 4) + IMD, employment status, Medication status | 1.13 (1.07-1.20) *p*<.001 | 1.13 (1.07-1.20) *p*<.001 | 0.72 (0.65-0.80) *p*<.001 | 0.86 (0.80-0.91) *p*<.001 |

## **Differences in treatment outcomes between minoritized ethnic males (using Office for National Statistics ethnicity categories) and White-British males, independent of particular patient characteristics and factors**

Table 24: RQ2.1 using ONS categories of ethnicity; White-British = reference category (male sub-group)

| **Model** | **Variables** | **ONS ethnicity categories** | **Reliable recovery: odds ratio (95% CI) *p*-value** | **Reliable improvement: odds ratio (95% CI) *p*-value** | **Reliable deterioration: odds ratio (95% CI) *p*-value** | **Drop-out: odds ratio (95% CI) *p*-value** |
| --- | --- | --- | --- | --- | --- | --- |
| Model 1 | Ethnicity | Asian  Black  Chinese  Mixed  Other  White-other | 0.67 (0.62-0.71) *p*<.001  0.73 (0.67-0.79) *p*<.001  1.03 (0.75-1.40) *p*=.000  0.79 (0.71-0.88) *p*<.001  0.60 (0.53-0.67) *p*<.001  0.82 (0.77-0.87) *p*<.001 | 0.77 (0.72-0.83) *p*<.001  0.84 (0.77-0.91) *p*<.001  0.83 (0.60-1.15) *p*=.273  0.93 (0.83-1.09) *p*=.197  0.70 (0.62-0.80) *p*<.001  0.88 (0.83-0.94) *p*<.001 | 1.52 (1.35-1.72) *p*<.001  1.33 (1.15-1.53) *p*<.001  1.18 (0.67-2.08) *p*=.575  1.08 (0.89-1.33) *p*=.415  1.72 (1.42-2.08) *p*<.001  1.19 (1.06-1.33) *p*=.003 | 1.41 (1.31-1.53) *p*<.001  1.60 (1.46-1.74) *p*<.001  0.90 (0.62-1.30) *p*=.573  1.26 (1.12-1.41) *p*<.001  1.36 (1.19-1.56) *p*<.001  1.02 (0.95-1.10) *p*=.539 |
| Model 2 | (Model 1) + Service | Asian  Black  Chinese  Mixed  Other  White-other | 0.63 (0.59-0.68) *p*<.001  0.72 (0.66-0.77) *p*<.001  1.04 (0.77-1.42) *p*=.793  0.80 (0.72-0.88) *p*<.001  0.60 (0.53-0.68) *p*<.001  0.83 (0.78-0.88) *p*<.001 | 0.76 (0.70-0.82) *p*<.001  0.83 (0.76-0.90) *p*<.001  0.83 (0.61-1.15) *p*=.263  0.93 (0.83-1.03) *p*=.172  0.70 (0.62-0.79) *p*<.001  0.88 (0.82-0.94) *p*<.001 | 1.57 (1.38-1.78) *p*<.001  1.31 (1.14-1.52) *p*<.001  1.21 (0.68-2.13) *p*=.521  1.09 (0,89-1.33) *p*=.410  1.73 (1.42-2.10) *p*<.001  1.19 (1.06-1.34) *p*=.002 | 1.32 (1.22-1.44) *p*<.001  1.58 (1.44-1.72) *p*<.001  0.99 (0.68-1.44) *p*=.962  1.20 (1.15-1.46) *p*<.001  1.49 (1.30-1.70) *p*<.001  1.11 (1.03-1.19) *p*=.005 |
| Model 3 | (Model 2) + Age, LTC status | Asian  Black  Chinese  Mixed  Other  White-other | 0.63 (0.58-0.68) *p*<.001  0.72 (0.66-0.78) *p*<.001  1.04 (0.76-1.43) *p*=.803  0.78 (0.54-0.70) *p*<.001  0.61 (0.54-0.70) *p*<.001  0.83 (0.78-0.88) *p*<.001 | 0.75 (0.69-0.81) *p*<.001  0.83 (0.76-0.90) *p*<.001  0.82 (0.59-1.14) *p*=.242  0.91 (0.81-1.03) *p*=.124  0.71 (0.62-0.80) *p*<.001  0.88 (0.82-0.94) *p*<.001 | 1.61 (1.41-1.83) *p*<.001  1.30 (1.12-1.52) *p*=.001  1.31 (0.74-2.38) *p*=.350  1.13 (0.92-1.39) *p*=.253  1.71 (1.39-2.10) *p*<.001  1.19 (1.06-1.34) *p*=.004 | 1.30 (1.20-1.42) *p*<.001  1.58 (1.44-1.73) *p*<.001  0.87 (0.59-1.28) *p*=.469  1.20 (1.07-1.36) *p*=.003  1.45 (1.25-1.67) *p*<.001  1.09 (1.02-1.18) *p*=.018 |
| Model 4 | (Model 3) + Baseline severity PHQ9, Baseline severity GAD7, Baseline severity WSAS, diagnosis/presenting problem, social phobia, agoraphobia, specific phobia | Asian  Black  Chinese  Mixed  Other  White-other | 0.78 (0.72-0.85) *p*<.001  0.87 (0.80-0.96) *p*=.005  1.14 (0.88-1.02) *p*=.489  0.92 (0.82-1.04) *p*=.193  0.77 (0.66-0.89) *p*<.001  0.92 (0.85-0.99) *p*=.022 | 0.79 (0.72-0.86) *p*<.001  0.91 (0.88-1.00) *p*=.052  1.04 (0.70-1.55) *p*=.834  0.99 (0.86-1.12) *p*=825  0.73 (0.63-0.85) *p*<.001  0.90 (0.83-0.97) *p*=.009 | 1.71 (1.48-1.96) *p*<.001  1.22 (1.03-1.45) *p*=.020  0.82 (0.39-1.71) *p*=.596  1.15 (0.91-1.44) *p*=.239  2.04 (1.62-2.56) *p*<.001  1.20 (1.05-1.38) *p*=.008 | 1.15 (1.05-1.27) *p*=.002  1.45 (1.21-1.61) *p*<.001  0.74 (0.46-1.17) *p*=.197  1.17 (1.02-1.34) *p*=.025  1.33 (1.13-1.56) *p*<.001  1.05 (0.96-1.14) *p*=.263 |
| Model 5 | (Model 4) + IMD, employment status, Medication status | Asian  Black  Chinese  Mixed  Other  White-other | 0.75 (0.69-0.82) *p*<.001  0.93 (0.84-1.03) *p*=.140  1.06 (0.72-1.56) *p*=.766  0.95 (0.84-1.08) *p*=.434  0.83 (0.71-0.96) *p*=.016  0.95 (0.88-1.02) *p*=.164 | 0.77 (0.70-0.84) *p*<.001  0.95 (0.86-1.06) *p*=.374  0.99 (0.65-1.50) *p*=.957  1.00 (0.87-1.15) *p*=.973  0.81 (0.69-0.94) *p*=.007  0.92 (0.85-1.00) *p*=.057 | 1.68 (1.15-1.96) *p*<.001  1.13 (0.94-1.36) *p*=.192  1.01 (0.48-2.13) *p*=.972  1.16 (0.92-1.48) *p*=.211  1.99 (1.56-2.53) *p*<.001  1.29 (1.11-1.49) *p*=.001 | 1.18 (1.07-1.30) *p*=.001  1.42 (1.28-1.58) *p*<.001  0.79 (0.49-1.27) *p*=.322  1.15 (1.00-1.32) *p*=.054  1.28 (1.09-1.51) *p*=.003  1.04 (0.95-1.13) *p*=.402 |

As with the female sub-sample, analyses also explored differences in outcomes between White-British males and males from different minoritized ethnic groups using the ONS ethnicity categories. When controlling for socio-demographic and clinical factors, least likely to recover when compared to White-British males, were Asian males (OR=0.75 [95% CI: 0.0.69-0.82)], *p*<.001), followed those in the ‘other’ ethnic group (OR= 0.83 [95% CI:0.71-0.96], *p*=.016).

Both Asian males and males in the ‘other’ ethnic group achieved significantly lower improvement than White-British counterparts (Asian: OR=0.77 [95% CI: 0.70-0.84] *p*<.001); (Other: OR=0.81 [95% CI: 0.69-0.94], *p*=.007) when controlling for socio-demographic and clinical factors.

Significantly increased odds of deterioration were observed for several minoritized ethnic groups when compared to White-British males. The largest figure was seen for males in the ‘other’ category (OR=1.99 [95% CI: 1.56-2.53] *p*<.001), followed by Asian males (OR=1.68 [95% CI: 1.15-1.96], *p*<.001) and White-other males (OR=1-29 [95% CI: 1.11-1.49), *p*=.001).

Black, ‘other’ and Asian males all showed significantly increased odds of drop-out compared to White-British counterparts (Black: OR=1.42[95% CI: 1.28-1.58] *p*=.001); (‘other’: OR=1.28 [95% CI: 1.09-1.51] *p*=.003); (Asian: OR=1.18 [95% CI: 1.07-1.30], *p*=.001).

### Employment and IMD

An additional analysis (Model 6, **Table 25**) was performed to explore the impact of removing IMD and employment as potential confounding variables, on reliable recovery outcomes.

Table 25: Linear regression model removing IMD and employment status as potential confounders (male subsample)

| **Model** | **Variables** | **ONS ethnicity categories** | **Reliable recovery: odds ratio (95% CI) *p*-value** |
| --- | --- | --- | --- |
| Model 5 | (Model 4) + IMD, employment status, Medication status | Asian  Black  Chinese  Mixed  Other  White-other | 0.75 (0.69-0.82) *p*<.001  0.93 (0.84-1.03) *p*=.140  1.06 (0.72-1.56) *p*=.766  0.95 (0.84-1.08) *p*=.434  0.83 (0.71-0.96) *p*=.016  0.95 (0.88-1.02) *p*=.164 |
| Model 6 | (Model 4) + Medication status | Asian  Black  Chinese  Mixed  Other  White-other | 0.75 (0.69-0.82) *p*<.001  0.86 (0.78-0.95) *p*=.002  1.13 (0.77-1.65) *p*=.545  0.92 (0.81-1.04) *p*=.176  0.77 (0.66-0.89) *p*=.001  0.92 (0.85-0.99) *p*=.032 |

Removal of IMD and employment status as variables in the model resulted in significantly lower odds of reliable recovery for Black and White-other males relative to White-British males (**Table 25**).

## **Organization-level factors**

Logistic regression models with interactions terms were used to explore the potential differential effects of organization-level factors on outcomes for different ethnic groups, whilst controlling for socio-demographic and clinical variables. For males, the only outcomes for which significant interactions between ethnic group and organization-level variables were seen, were for reliable recovery and drop-out.

Table 26: Results from the likelihood-ratio test used to compare the adjusted model (model 5) with the inclusion of an interaction term

| **Organization-level variable** | **Reliable recovery** | **Reliable improvement** | **Deterioration** | **Dropout** | |
| --- | --- | --- | --- | --- | --- |
| Number of sessions | *X^2^* (6) = 11.73 *p*=.068 | *X^2^* (6) = 7.85, *p*=.249 | *X^2^* (6) = 7.92, *p*=.244 | *X^2^* (6) = 21.30, *p*=.002 | |
| Referral source (GP/self-referral) | *X^2^* (11) = 14.89, *p*=.188 | *X^2^* (11) = 10.20, *p*=.512 | *X^2^* (11) = 6.01, *p*=.872 | *X^2^* (11) = 14.92, *p*=.186 | |
| Number of face-to-face sessions | *X^2^* (6) = 12.12 *p*=.059 | *X^2^* (6) = 7.13, *p*=.308 | *X^2^* (6) = 8.85, *p*=.182 | *X^2^* (6) = 16.25, *p*=.013 | |
| Number of telephone sessions | *X^2^* (6) = 4.15, *p*=.656 | *X^2^* (6) = 4.62, *p*=.594 | *X^2^* (6) = 1.82, *p*=.308 | *X^2^* (6) = 4.17, *p*=.654 | |
| Number of video call sessions | *Omitted | *Omitted | *Omitted | *Omitted | |
| Number of days from referral to assessment (primary wait) | *X^2^* (6) = 15.46, *p*=.486 | *X^2^* (6) = 2.17, *p*=.903 | *X^2^* (6) = 5.35, *p*=.308 | *X^2^* (6) = 14.33, *p*=.026 | |
| Number of days assessment to treatment (secondary wait) | *X^2^* (6) = 1.76, *p*=.940 | *X^2^* (6) = 3.42, *p*=.755 | *X^2^* (6) = 4.87, *p*=.560 | *X^2^* (6) = 5.36, *p*=.496 | |
| Number of high intensity treatment sessions | *X^2^* (6) = 8.30, *p*=.217 | *X^2^* (6) = 3.67, *p*=.721 | *X^2^* (6) = 5.53, *p*=.478 | *X^2^* (6) = 8.67 *p*=.193 | |
| **Missing values led to unequal observations between models, restricting comparison using the likelihood-ratio test.* | | | | |  |

Inclusion of interaction terms resulted in improvement in several of the models (**Table 26**). The results from the improved models (i.e., *p*<.005) are provided below.

### **Reliable recovery**

Table 27: Results from logistic regression models with interactions for the male subsample – reliable recovery outcome. Included are the factors for each ethnic group that resulted in odds ratios with a p-value of <.05. (reference category = White-British males)

| **Reliable recovery** | |
| --- | --- |
| **Organization-level variables** | **Ethnic group** |
|  | **Black** |
| Number of sessions | OR=1.03 (CI:1.00-1.05), *p*=.028 |
| Form used to provide treatment (number of face to face treatment sessions) | OR=1.03 (CI: 1.00-1.05), *p*=.009 |

There was a small but significant interaction between specific ethnicity groups and the number of treatment sessions received. Specifically, a higher number of sessions was associated with a small increase in the odds of reliable recovery for Black males relative to White-British males (OR=1.03 [95% CI:1.00-1.05], *p*=.028).


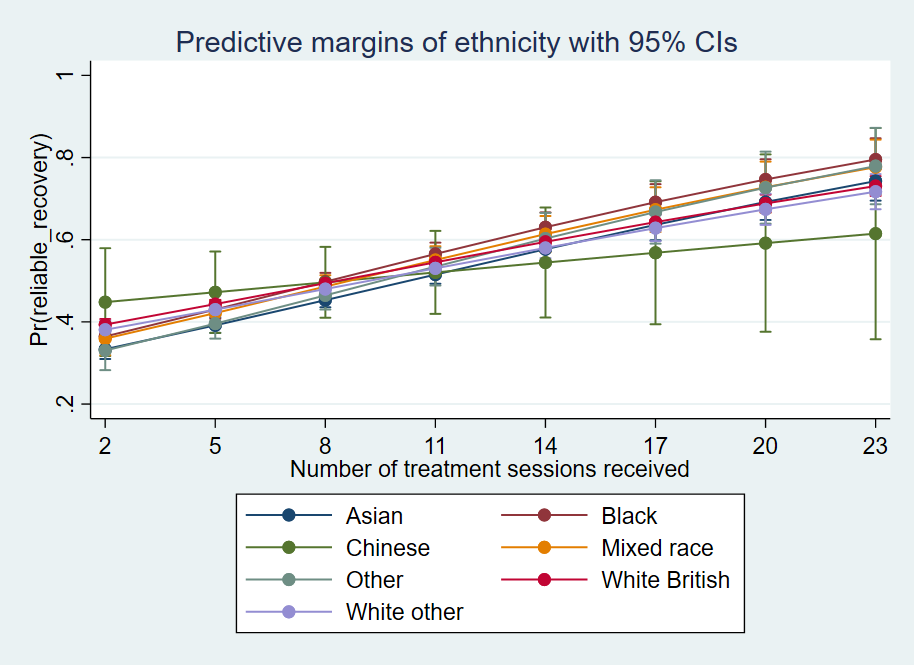


Figure 22: Graph to show ethnic group and number of treatment sessions received as predictors of reliable recovery (male sub-sample)

The number of face-to-face treatment sessions was associated with a small significant increase in odds of reliable recovery for males in the Black ethnicity group, relative to White British males (OR=1.03 [95% CI: 1.00-1.05) *p*=.009).


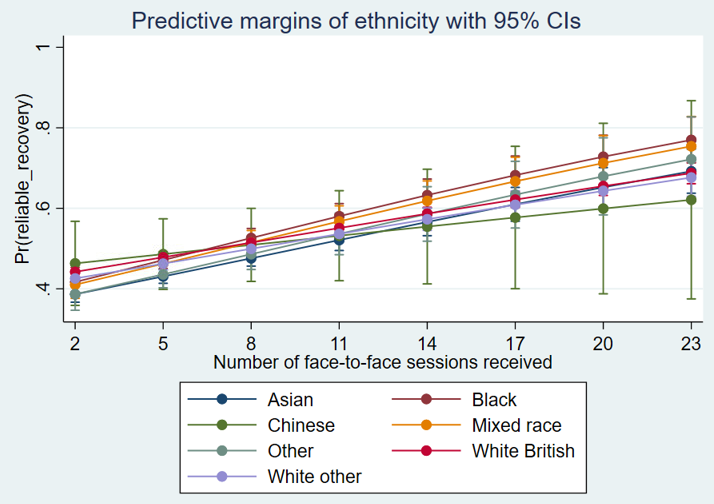


Figure 23: Graph to show ethnic group and number of face-to-face treatment sessions received as predictors of reliable recovery (male sub-sample)

### **Drop-out**

Table 28: Results from logistic regression models with interactions for the male subsample – dropout outcome. Included are the factors for each ethnic group that resulted in odds ratios with a p-value of <.05. (reference category = White-British males

| **Drop-out** | |
| --- | --- |
| **Organization-level variables** | **Ethnic group** |
|  | **Asian** |
| Number of sessions | OR=0.93 (CI: 0.89-0.97), *p*=.001 |
| Form used to provide treatment (number of face to face treatment sessions) | OR=0.95 (CI: 0.92-0.98), *p*=.001 |
| Number of high intensity treatment sessions | OR=0.97 (CI: 0.94-0.99), *p*=.011 |

The number of treatment sessions was associated with a small significant decrease in odds of drop-out for males in the Asian males, relative to White-British males (OR=0.93 [95% CI: 0.89-0.97], *p*=.001).


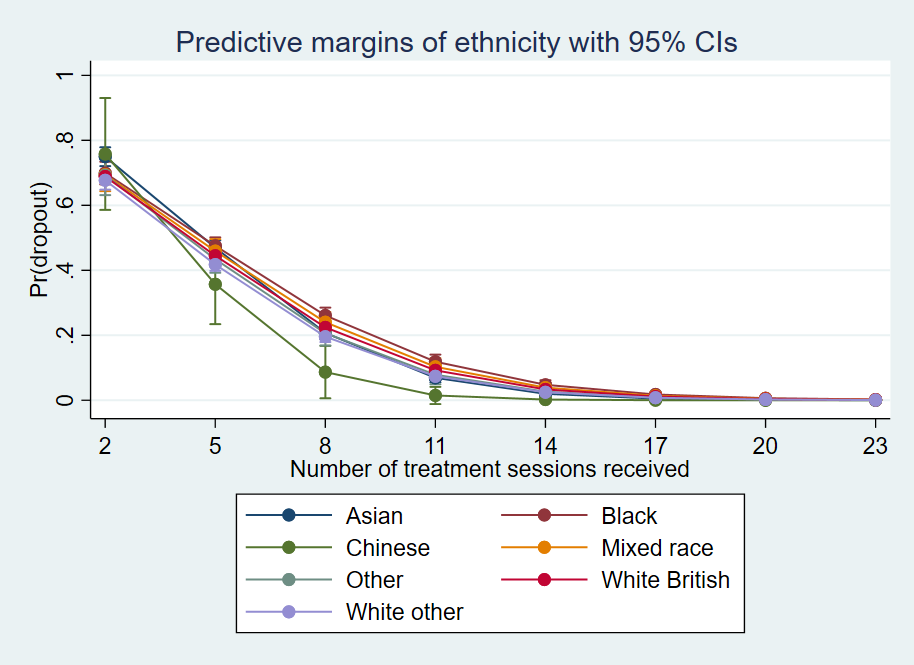


Figure 24: Graph to show ethnic group and number of treatment sessions received as predictors of drop-out (male sub-sample)

A similar association was observed regarding the number of high intensity sessions received, which resulted in decreased odds of drop-out for Asian males relative to White-British males (OR=0.97 [95% CI: CI:0.94-0.99], *p*=.011).


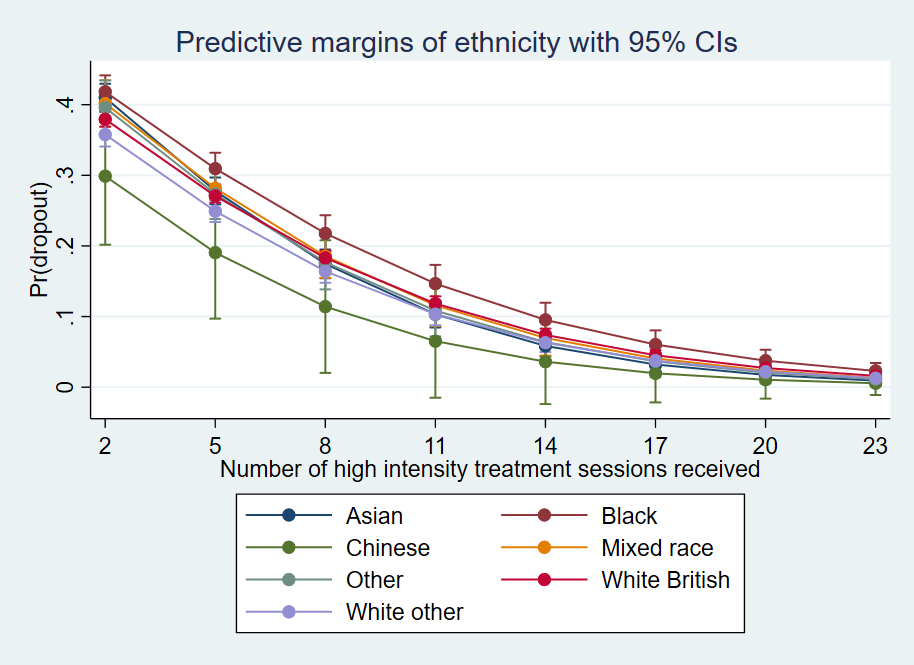


Figure 25: Graph to show ethnic group and number of high intensity treatment sessions received as predictors of drop-out (male sub-sample)

The number of face-to-face treatment sessions was associated with a small significant decrease in odds of drop-out for males in the Asian males, relative to White-British males (OR=0.95 [95% CI:0.92-0.98], *p*=.001).


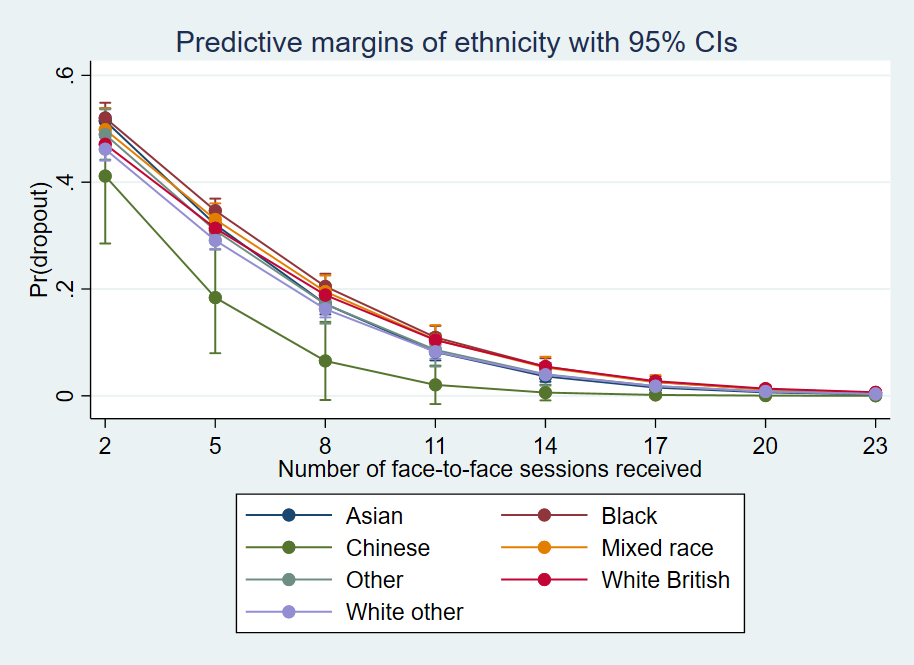


Figure 26: Graph to show ethnic group and number of face-to-face treatment sessions received as predictors of drop-out (male sub-sample)
